# Human extracellular microvesicles from renal tubules reverse kidney ischemia-reperfusion injury in rats

**DOI:** 10.1371/journal.pone.0202550

**Published:** 2018-08-27

**Authors:** James M. Dominguez, Jesus H. Dominguez, Danhui Xie, K. J. Kelly

**Affiliations:** 1 Department of Medicine, Indiana University School of Medicine, Indianapolis, IN, United States of America; 2 Roudebush VA Medical Center, Indianapolis, IN, United States of America; Universita degli Studi di Torino, ITALY

## Abstract

Hypoxic acute kidney injury, a major unresolved problem, initiates, or aggravates, renal functional and structural decline. There is no treatment for hypoxic acute renal injury and its sequelae. We tested the hypothesis that human kidney tubular cells, or their extracellular vesicles (exosomes), prevent renal injury when infused intravenously 24 hours after 50 minutes of bilateral renal ischemia in Nude rats. Cells and their exosomes were from harvested human kidneys declined for transplantation. Injections of either cells or exosomes, given after 24 and 48 hours of reperfusion, preserved renal function and structure in both treatment groups. However, exosomes were superior to cells; and maintained renal vascular and epithelial networks, prevented renal oxidant stress, and apoptosis; and restrained activation of pro-inflammatory and pro-fibrogenic pathways. Exosomes worked in 24 hours, consistent with functional rather than regenerative activity. Comprehensive proteomic analysis identified 6152 renal proteins from all cellular compartments; and 628 were altered by ischemia at all cell levels, while 377 were significantly improved by exosome infusions. We conclude that renal damage from severe ischemia was broad, and human renal exosomes prevented most protein alterations. Thus, exosomes seem to acutely correct a critical and consequential abnormality during reperfusion. In their absence, renal structure and cells transition to a chronic state of fibrosis and extensive renal cell loss.

## Introduction

Hypoxic acute kidney injury (AKI) is a syndrome linked to high morbidity and mortality [1, 2]. AKI aggravates chronic renal failure (CRF) [3], and accelerates the decline to end-stage renal disease (ESRD) [4]. There is no treatment for AKI, a complicated and unpredictable syndrome characterized by broad and devastating renal dysfunction [5]. Multiple attempts to treat AKI have failed, and it is recognized that an entirely different therapeutic approach is needed [5]. We have previously reported that infusions of adult rat renal tubular cells prevented renal injury, in that transplanted cells improved function and structure in diverse rat models of acute and chronic renal injury [6–10]. We also recognized an apparent paradox in our data, as a relatively small number of infused cells had broad beneficial renal effects [11]. Hence, we hypothesized that transplanted primary renal cells amplified their actions through released extracellular vesicles (EVs) in situ [12].

Exosomes, the best characterized type of EVs; are secreted nanovesicles (30–150nm in diameter) that contain proteins, lipids, mRNA and miRNAs [12]. Secreted exosomes interconnect cells, and convey the metabolic state of the originating cells, including protective responses to hypoxia [13–15]. For example, we have showed that renal exosomes released from hypoxia pre-conditioned renal tubular cells (i.e., HPC exosomes) prevented most manifestations of severe AKI [11]. We now compared the effects of HPC human kidney tubular cells with their exosomes on athymic Nude rats with severe hypoxic AKI. We found that after 24 hours of reperfusion, peripheral intravenous infusion of human kidney tubular cells, or their exosomes, protected severely post-ischemic kidneys at multiple levels, including structure, function and expressed proteins.

## Methods

Complete description of methods can be found in the Supplemental section.

## Results

### Human renal tubular cells and HLA A1 expression in rat kidneys

**Fig 1:**
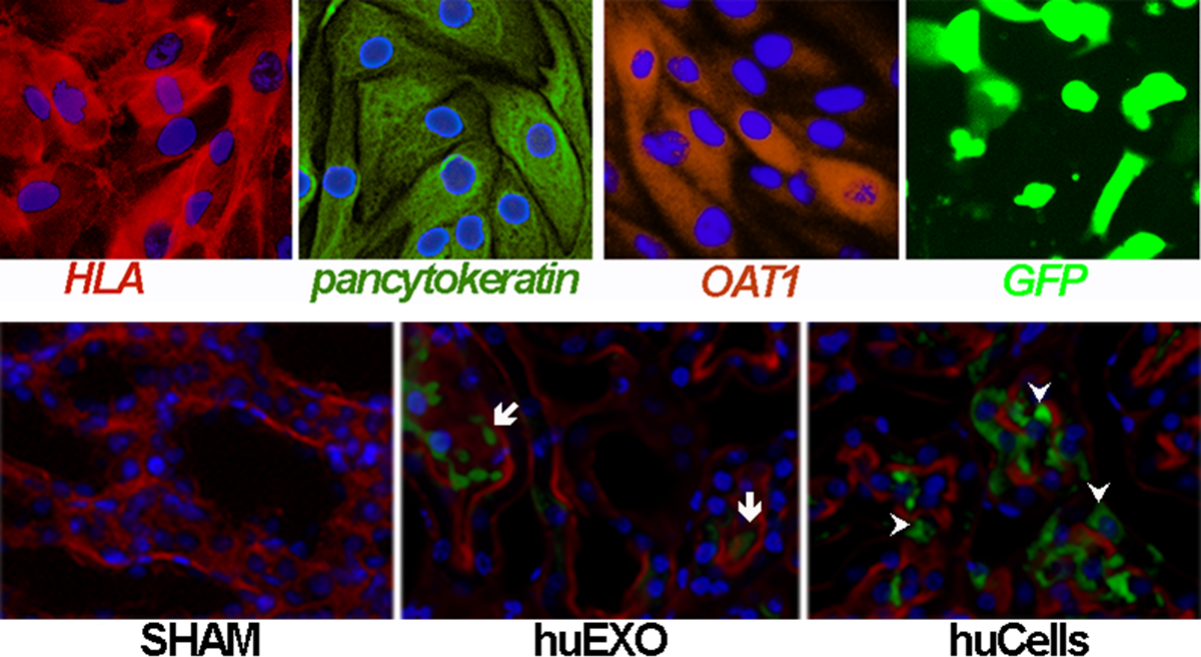
Human renal cells and HLA A1 expression in rat kidneys. Top, human kidney tubular cells were 99 ± 1% positive for HLA-A1 (red, left), 99 ± 1% positive for Pan-cytokeratin (green, second from left), 77 ± 4% positive for OAT1 (red, second from right), and 63 ± 3% positive for transfected GFP (right), n = 4. Bottom, HLA-A1 immunoreactivity (green) was not detected in sham rats (SHAM; left). In contrast, HLA-A1 (green) was expressed in renal tubules of ischemic rats injected with kidney cell exosomes (huEXO; middle, arrows), or their originating transplanted cells (huCELLS, right, arrow heads). Renal F actin was stained with rhodamine phalloidin (red) to outline cells, and Hoechst 33342 (blue) was added to label nuclei.

Cultured human renal tubular cells expressed key epithelial features prior to infusion, Fig 1. The donor cells were positive for Human Leukocyte Antigen A1 and for pan-cytokeratin, consistent with their human epithelial derivation. They were 77 ± 4% positive for the organic anion transporter 1, a renal proximal tubule marker. We presume the remaining epithelial cells were from other nephron segments or had precursor cell background [16], although they did not express the stem cell marker nanog. In addition, they did not express endothelial markers CD31 or E-selectin or glomerular podocyte markers nephrin or synaptopodin or the fibroblast markers alpha smooth muscle actin or fibroblast specific protein-1. Cells were also transfected with a green fluorescent protein (GFP) plasmid prior to infusion, and expressed GFP. In addition, HLA immunoreactivity was mainly found in renal tubules of rats injected with human cells or their exosomes, but not in glomeruli. HLA was not detected in non-injected sham rats.

### Characterization of human renal exosomes

**Fig 2:**
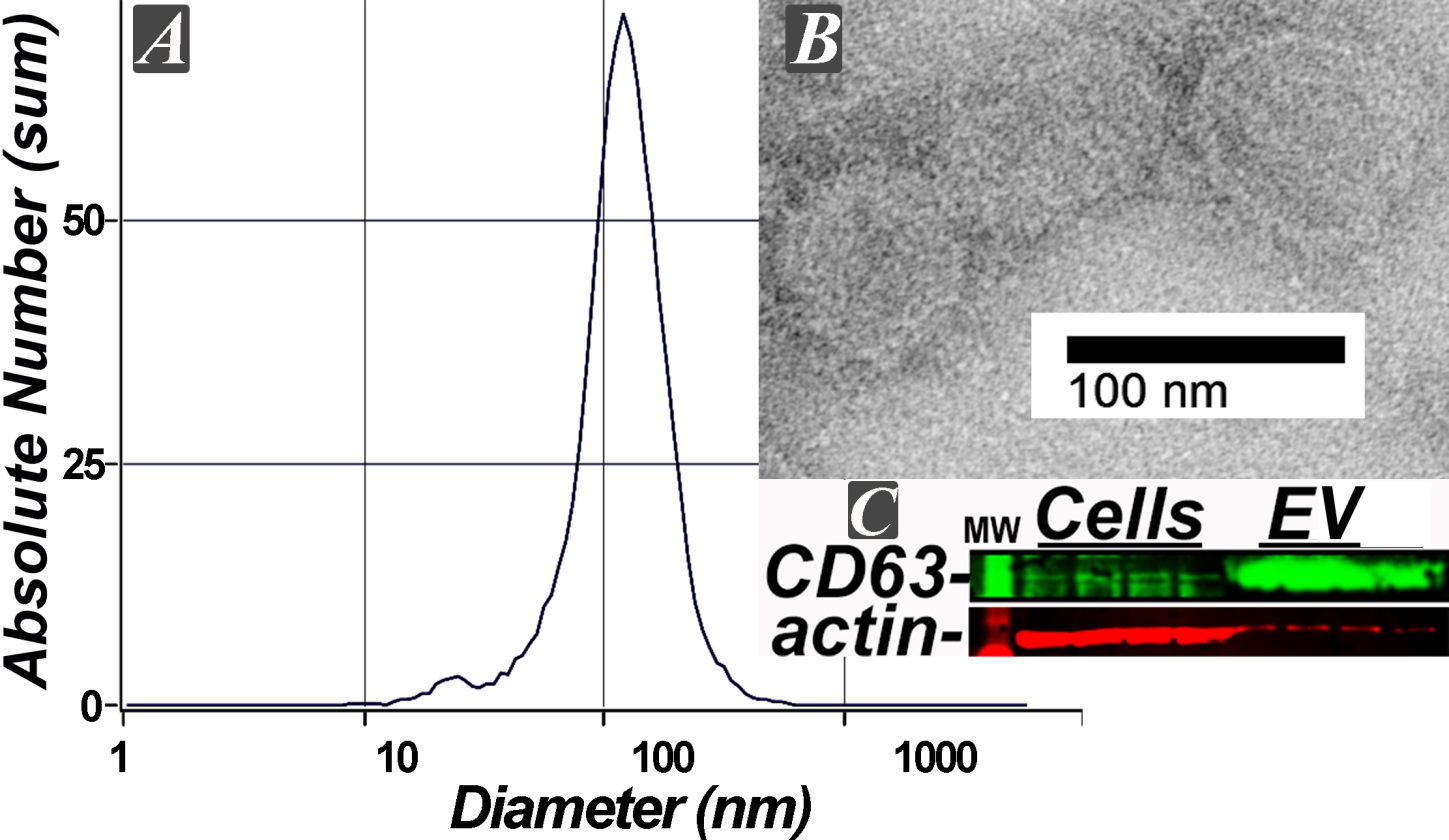
Characterization of human exosomes. A, the median diameter of exosomes measured by a nanoparticle analyzer was 115 ± 0.9 nM; n = 5 separate samples. B, electron microscopy of isolated exosomes verifies diameter size measured by the particle analyzer. C, western blot showing enrichment of CD63 (green), an exosomal protein marker, when compared to the originating cells, n = 4.

We used multiple and independent assays to characterize human renal exosomes prior to infusion, Fig 2 [17]. The median diameter of exosomes measured by nano particle tracker was 115 ± 0.9 nM (n = 5), which is consistent with the size of exosomes, and no other vesicles [17]. This vesicle size was further verified by electron microscopy. In addition, isolated renal exosomes were enriched with the membrane exosome marker CD63.

### Renal localization of injected cells and exosomes

**Fig 3:**
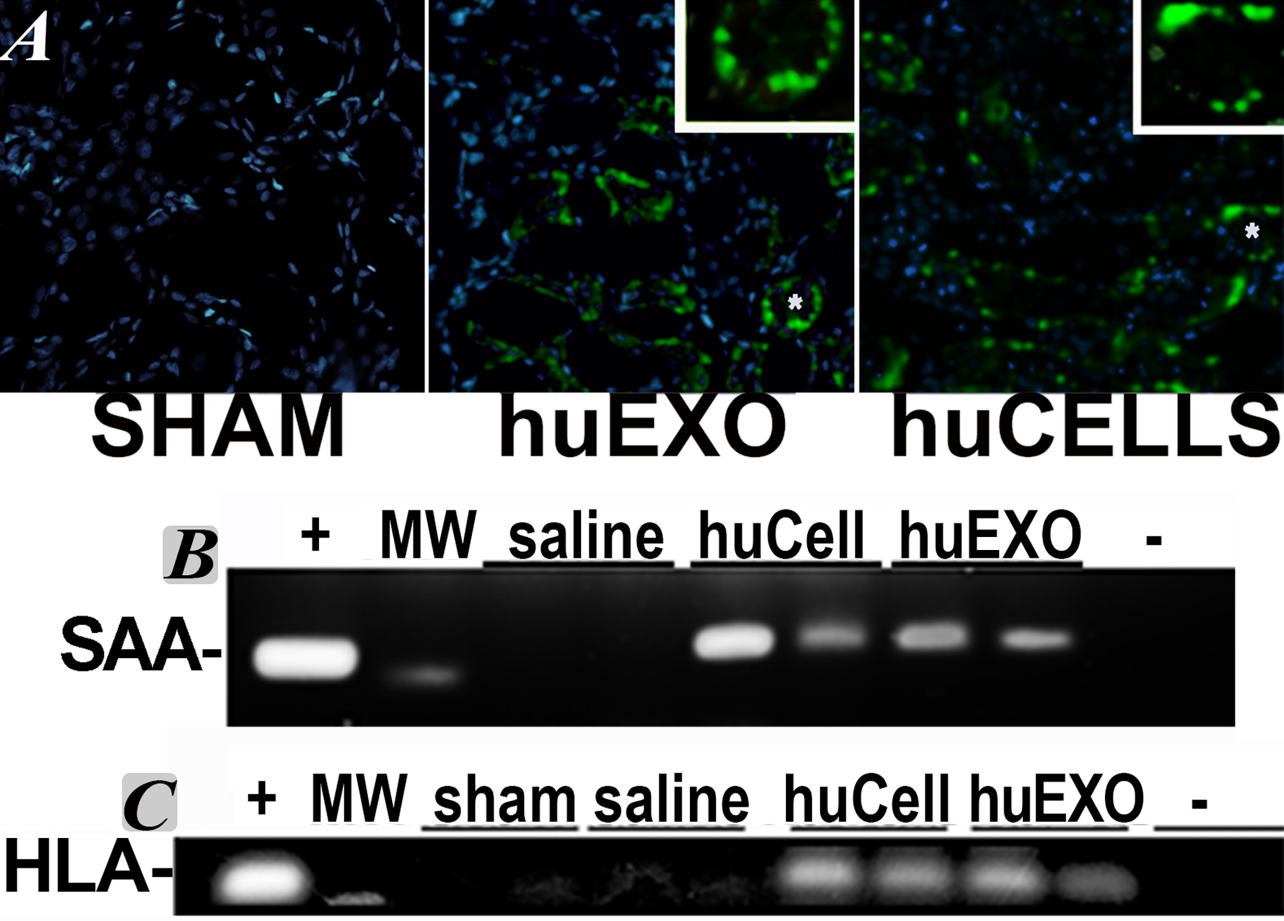
Localization of injected human exosomes and tubular cells in rat ischemic kidneys. A, representative frozen kidney sections of sham rats (SHAM, left), ischemic rats injected with human kidney exosomes labeled with Exo-Green Exo-Glow dye (huEXO, middle), or ischemic rats injected with GFP positive renal tubular cells (huCELLS, right). The rats were injected 24 hours after surgery; and exosomes or cells (green), were found in renal tubules. The insets are enlargements of tubules marked by asterisks. B, SAA1.3 transcript amplified by PCR, from pcDNA3.1-SAA1.3, plasmid positive control template (+). Amplification was also observed in kidneys of ischemic rats injected with kidney cells (huCell) and their exosomes (huEXO). SAA1.3 mRNA was absent in kidneys of ischemic rats injected with saline control and in the negative water control (-). C, human HLA-1A transcript amplified by PCR in normal human kidney cells, positive control (+), and from kidneys of ischemic rats injected with kidney cells (HuCell), or their exosomes (HuEXO). HLA-1A was absent in non-injected rats (sham) and in ischemic rats injected with saline vehicle, or in the negative water control (-).

Some exosomes were labeled with fluorescent Exo-glow before injection in the tail vein, and then were found localized in recipient ischemic kidneys [18]. We also found the originating human renal kidney tubular cells, expressing GFP, in the recipient rat kidneys after tail vein injection. Further verification of renal localization was achieved by PCR amplification of the human transcripts serum amyloid A 1.3 (SAA1) and human HLA-A1 mRNAs in rat kidneys. These transcripts were used to track injected cells and their exosomes in recipient rat kidneys, Fig 3. Specifically, human mRNA amplification occurred in kidneys that received human cells or their exosomes, but not in untreated sham rat kidneys. HLA-A1 transcripts were detected in isolated human kidney cells, and in kidneys of rats injected with human kidney cells or their exosomes, but not in kidneys of sham rats or post-ischemic rats injected with saline vehicle.

### Protection of renal function

**Fig 4:**
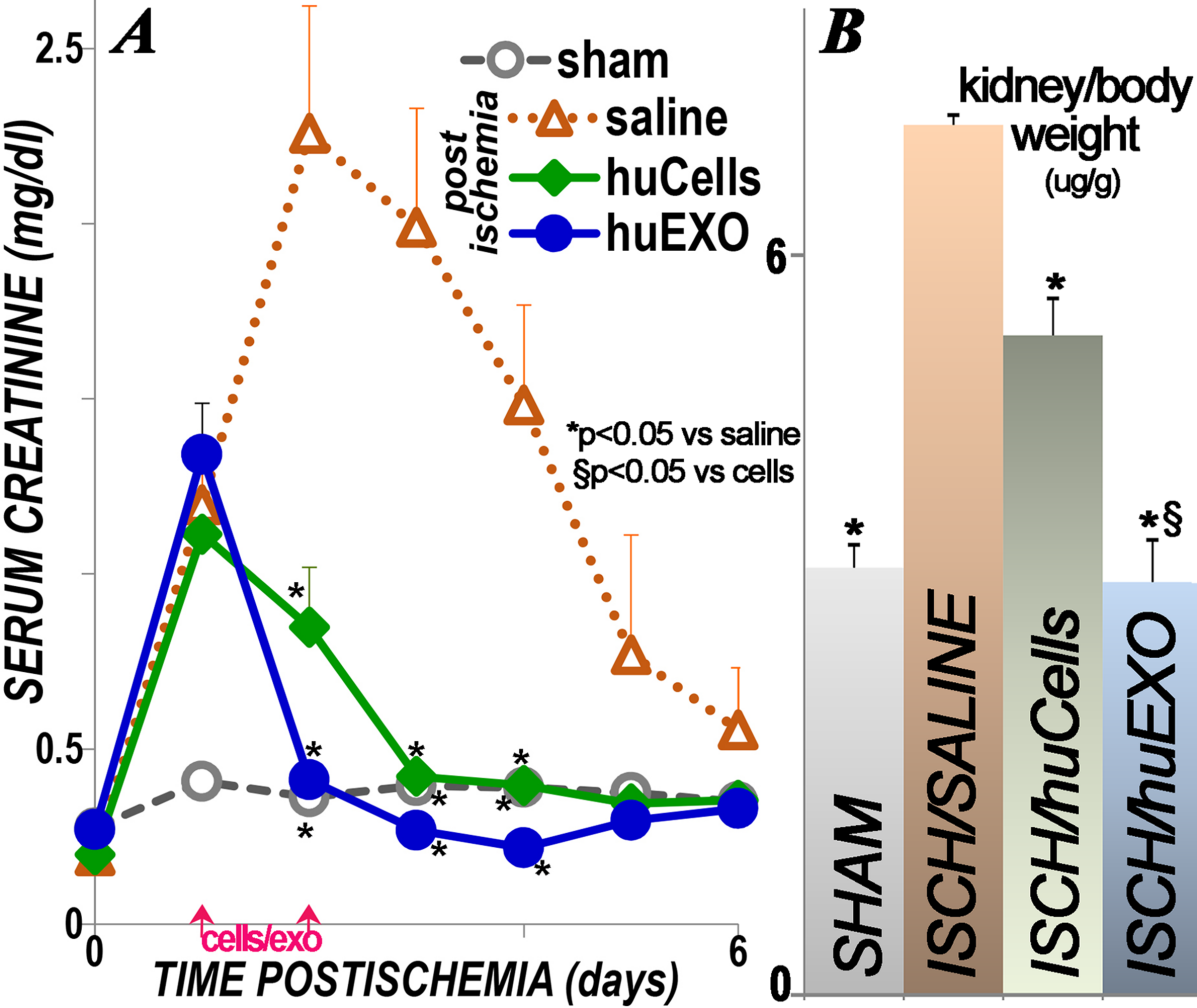
Renal function and renal size. A, serum creatinine levels in the four groups of rats, n = 4–5. Sham (sham, open circles), untreated ischemic (open triangles, saline), ischemic rats treated with human kidney cells (green diamonds, huCells) and ischemic rats treated with human exosomes (filled blue circles, huEXO). Time zero designates renal clamping, and cells, exosomes or saline vehicle were injected 1 and 2 days post-ischemia. (*) indicates significantly different than untreated ischemic rats (saline; p< 0.05). At 48 hours post-ischemia, HuEXO group was significantly lower than HuCells group (p <0.04). B, kidney weights were corrected for body weight. (*) indicates significantly lower than untreated ischemic rat group (ISCH/SALINE; p < 0.05). (§) indicates significantly lower than ischemic rat group injected with kidney cells (ISCH/huCells, p <0.05).

Renal function in Nude rats was estimated from measurements of serum creatinine, Fig 4. The data were collected over 6 days in all rats, sham surgery (n = 4), ischemic untreated (n = 4), ischemic treated with human renal cell transplants (3 X 10^**6**^ cells per intravenous injection, n = 5), and ischemic treated with human renal cell exosomes (13.6 E10 exosomes per intravenous injection, or 100 ug protein, n = 5).

Pre-ischemia mean serum creatinine level was similar in all groups; it increased rapidly in the three ischemic rat groups after 24 hours of reperfusion, and creatinine peaked at 48 hours of reperfusion in untreated ischemic rats. On the other hand, rats injected with human exosomes normalized their serum creatinine at 48 hours of reperfusion: 0.41 ± 0.02 mg/dl, as compared to sham rats, 0.36 ± 0.02, p = 0.25. Post-ischemic rats injected with human kidney cells exhibited a lag time at 48 hours of reperfusion, in that their average creatinine was 0.85 ± 0.17, or double that of sham or exosome treated ischemic rats, p = 0.04. Nevertheless, cell-treated ischemic rats still showed significant correction when compared to untreated ischemic rats at 48 hours, 2.26 ± 0.36, p = 0.02. Serum creatinine in untreated ischemic rats slowly declined during the six days of reperfusion, but it remained higher than the treated ischemic groups (p < 0.05, Fig 3). Post-mortem kidney weight in the untreated ischemia group was 40% higher than sham rats, 14% higher in the cell treated ischemia group, whereas the exosome treated group was indistinguishable from the sham group (Fig 3).

### Protection of renal structure and from apoptosis

**Fig 5:**
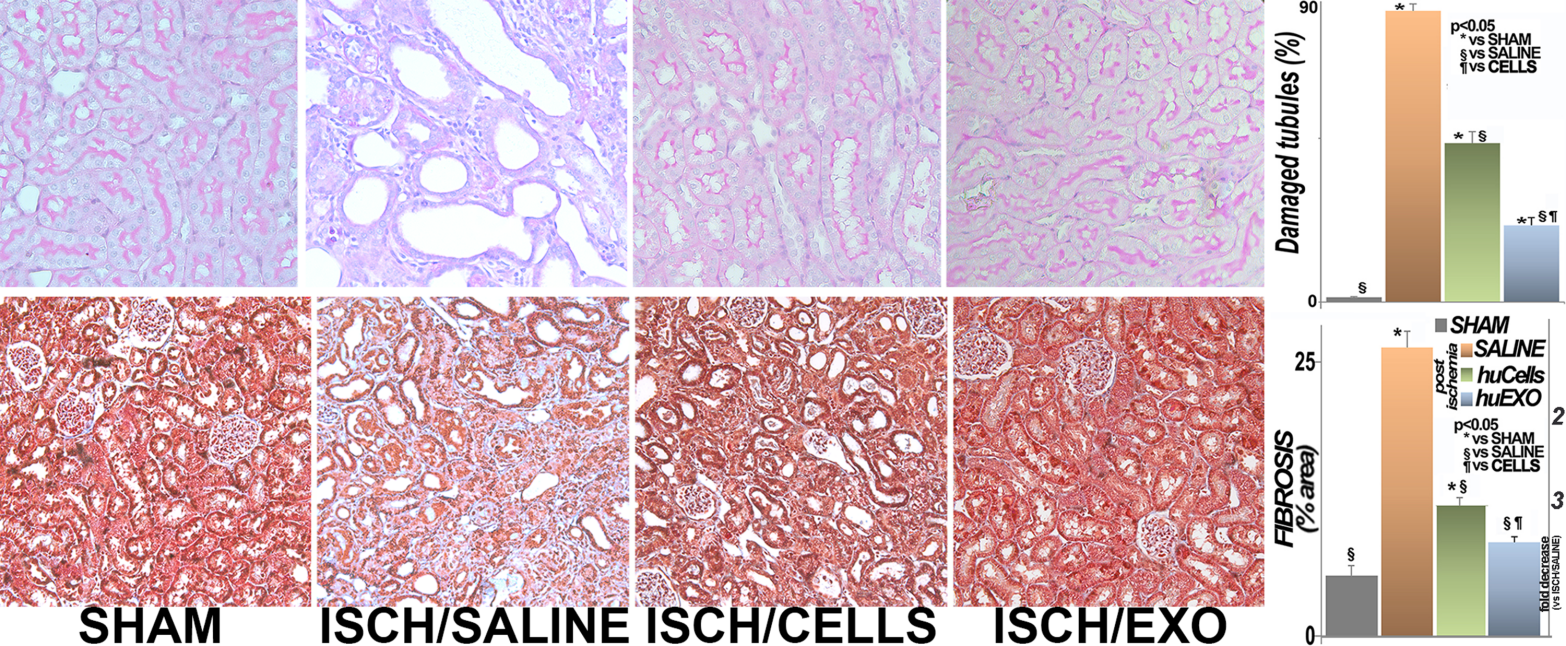
Renal structure. Top, representative images of renal sections stained with Periodic acid Schiff (PAS). Left to right, sham rats (SHAM), ischemic untreated rats (ISCH/SALINE), ischemic rats injected with kidney cells (ISCH/CELLS), and ischemic rats injected with their exosomes (ISCH/EXO). Tubular damage was widespread in untreated ischemia, including cellular attenuation and tubular dilation. These changes were improved in ISCH/CELLS, and largely prevented in ISCH/EXO. The data are summarized in the graph (right panel): (*), significantly different than sham group (SHAM; p < 0.05). (§), significantly lower than untreated ischemia rat group (ISCH/SALINE; p < 0.05. (¶), significantly lower than cell injected ischemia rat group (ISCH/CELLS, p < 0.05) (n = 27 measurements in each group). Bottom, representative images of renal sections stained with Masson’s trichrome. Interstitial renal fibrosis (blue) was extensive and replaced normal epithelia (red) in untreated ischemia (ISCH/SALINE), when compared to controls (SHAM). Treatment of renal ischemia with kidney cell injections (ISCH/CELLS) partly preserved the tubular epithelia, while injections with exosomes (ISCH/EXO) appeared to be more protective. The data are summarized in the graph (right panel): (*), significantly higher than sham controls (SHAM). (§), significantly lower than untreated ischemia rat group (ISCH/SALINE; p < 0.05). (¶), significantly lower than ischemia rat group injected with cells (ISCH/CELLS), p < 0.05. (n = 15 measurements in each group).

Renal pathological changes in all three groups of ischemic rats were compared to control (SHAM) rats at termination, i.e., at 6 days of reperfusion. The renal histology was evaluated in kidney sections stained with Periodic Acid Schiff (PAS) and with Masson’s trichrome, Fig 5.

**Fig 6:**
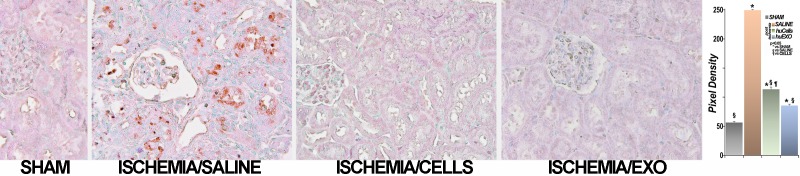
Representative renal images of TUNEL assay. Tubular apoptosis was extensive in untreated ischemic rats (brown, ISCHEMIA/SALINE), while it was nearly undetectable in control rats (SHAM). Treatment with either renal cells (ISCHEMIA/CELLS) or their exosomes (ISCHEMIA/EXO) prevented detectable post-ischemia apoptosis. The bar diagram summarizes this analysis: (*), significantly different than sham rat group (SHAM), p < 0.05. (§), significantly different than untreated ischemia group (SALINE), p < 0.05. (¶), significantly different than exosome infused group (huEXO), p < 0.05 (n = 16 measurements for SHAM, group; 32 measurements for untreated ischemia, SALINE, group, 16 measurements for cell treated ischemia group, huCells, and 20 measurements for exosome treated group, huEXO).

The ischemic kidneys exhibited marked tubular atrophy, attenuation and dilation. The pronounced tubular loss in untreated ischemia was accompanied by widespread interstitial fibrosis and cellular infiltrates. These ischemic changes were limited by cell transplantation, and tubular architecture was nearly intact in rats treated with renal exosomes. Tubular loss was also linked to tubular apoptosis, as previously reported [19], and it was far greater than in sham rats, or in ischemic rats treated with cells or with their exosomes, Fig 6.

### Protection of renal inflammation and microvascular damage

**Fig 7:**
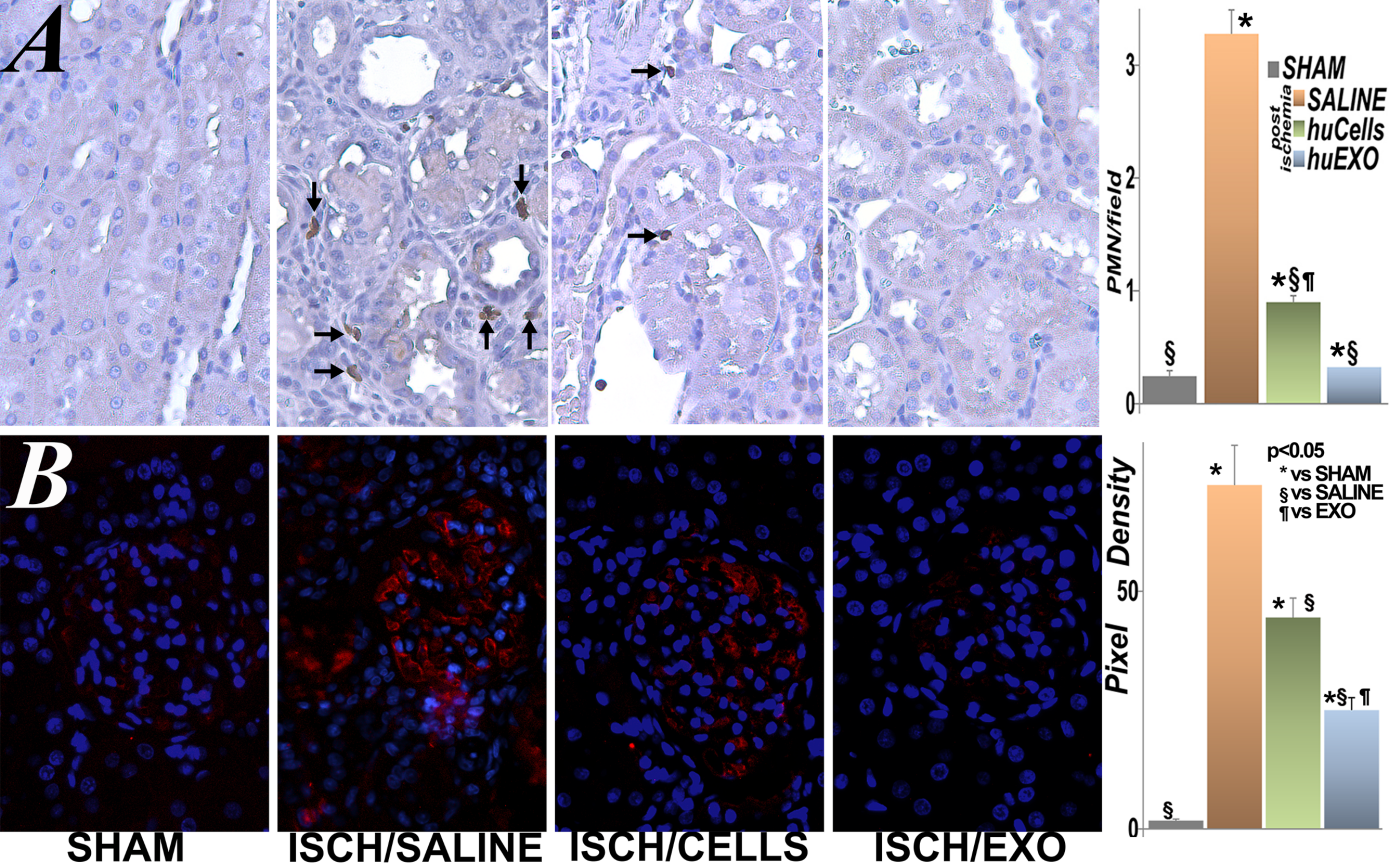
Renal inflammation. A, representative images of renal sections labeled with anti-neutrophil antibody. Neutrophils (brown) were detected in interstitial and intraluminal spaces of kidneys from untreated ischemic rats (ISCH/SALINE), while were barely seen in kidneys of sham rats (SHAM). In contrast, ischemic rats treated with kidney cells (ISCH/CELLS) or with exosomes (ISCH/EXO) had lower number of neutrophils. The bar diagram shows neutrophil number per 400X field (PMN/field): SHAM, SALINE, huCells, huEXO. (*), significantly higher than sham rats (SHAM), p< 0.05. (§), significantly lower than untreated ischemia (SALINE), p <0.05. (¶), significantly higher than ischemia treated with exosomes, huEXO, p < 0.05. (n = 67 measurements in each group). B, glomerular complement C3 expression (red) in sham rats was nearly undetectable, while it was heavily expressed in untreated ischemic rat kidneys. In contrast, treatment with renal cells or their exosomes limited C3 expression. Nuclei were counterstained with Hoechst (blue). The bar diagram shows average pixel density of C3 immunofluorescence per 400X power field. (*), significantly higher than sham rat group (SHAM), p< 0.05. (§), significantly lower than untreated ischemia rat group (SALINE), p <0.05. (¶), significantly higher than exosome injected ischemia rat group, EXO, p < 0.05. (n = 17 measurements in each group).

Renal ischemia causes renal inflammation, which is linked to functional decline [20, 21], and it seems to persist after the injury, although the entire process is not well characterized [22]. We found that immune deficient Nude rats [23] expressed unequivocal indicators of severe inflammation post-ischemia; presence of renal neutrophils infiltration and C3 complement component activation [24]. In marked contrast, post-ischemic rats infused with renal tubular cells, or their exosomes, had minimal increases in renal neutrophils and C3 activation, Fig 7.

### Protection of renal microvasculature

**Fig 8:**
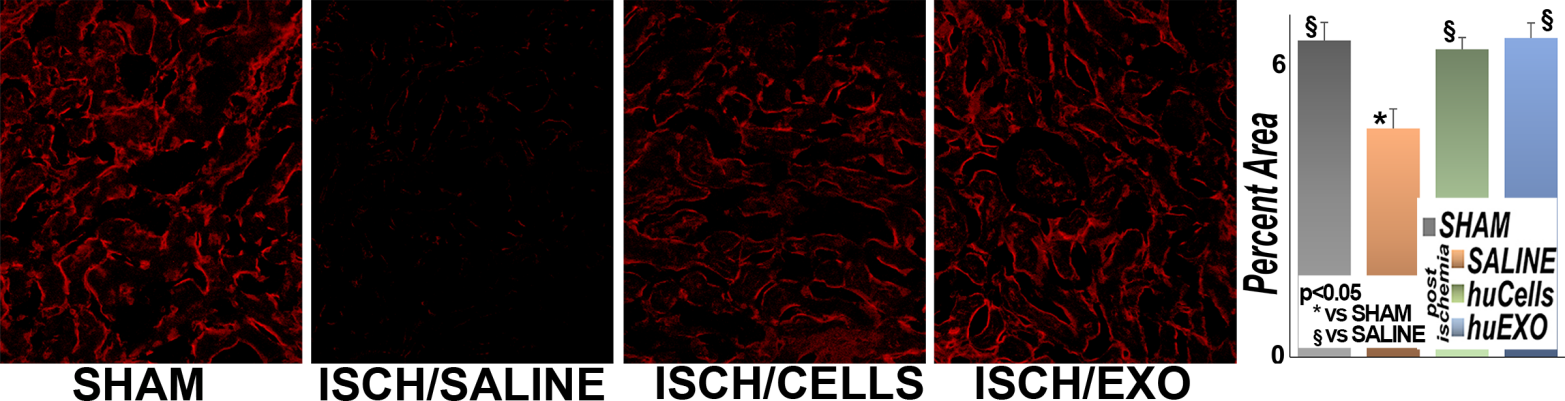
Renal microvasculature. Representative images of renal peritubular microvasculature labeled with DyLight 594 conjugated Lycopersicum esculentum (tomato lectin, LEL, red). The microvasculature was far less dense in untreated ischemia (SALINE) than in sham (SHAM). However, treatment with kidney cells (ISCH/CELLS) or their exosomes (ISCH/EXO) protected the microvasculature density and organization. The bar diagram shows the average fractional (fluorescence) positivity. (*), significantly lower than sham rat group (SHAM), p< 0.05. (§), significantly higher than untreated ischemia group (SALINE), p <0.05. (n = 115 measurements per group).

Renal ischemia also damages the microvasculature, resulting in sustained hypoxia, interstitial fibrosis and eventual chronic renal failure [25]. Accordingly, we investigated the role of cell and exosome infusions on the renal microvasculature in frozen sections stained with Lycopersicum esculentum lectin, Fig 8. As anticipated, the renal microvasculature was well-organized, and inter-connected in control sham rats. In contrast, untreated renal ischemia caused broad disruption and loss of renal micro vessels. However, ischemic rats infused with human kidney tubular cells, or their exosomes had well preserved renal microvasculature and similar to control rats.

### Protection of anti-oxidant defenses

**Fig 9:**
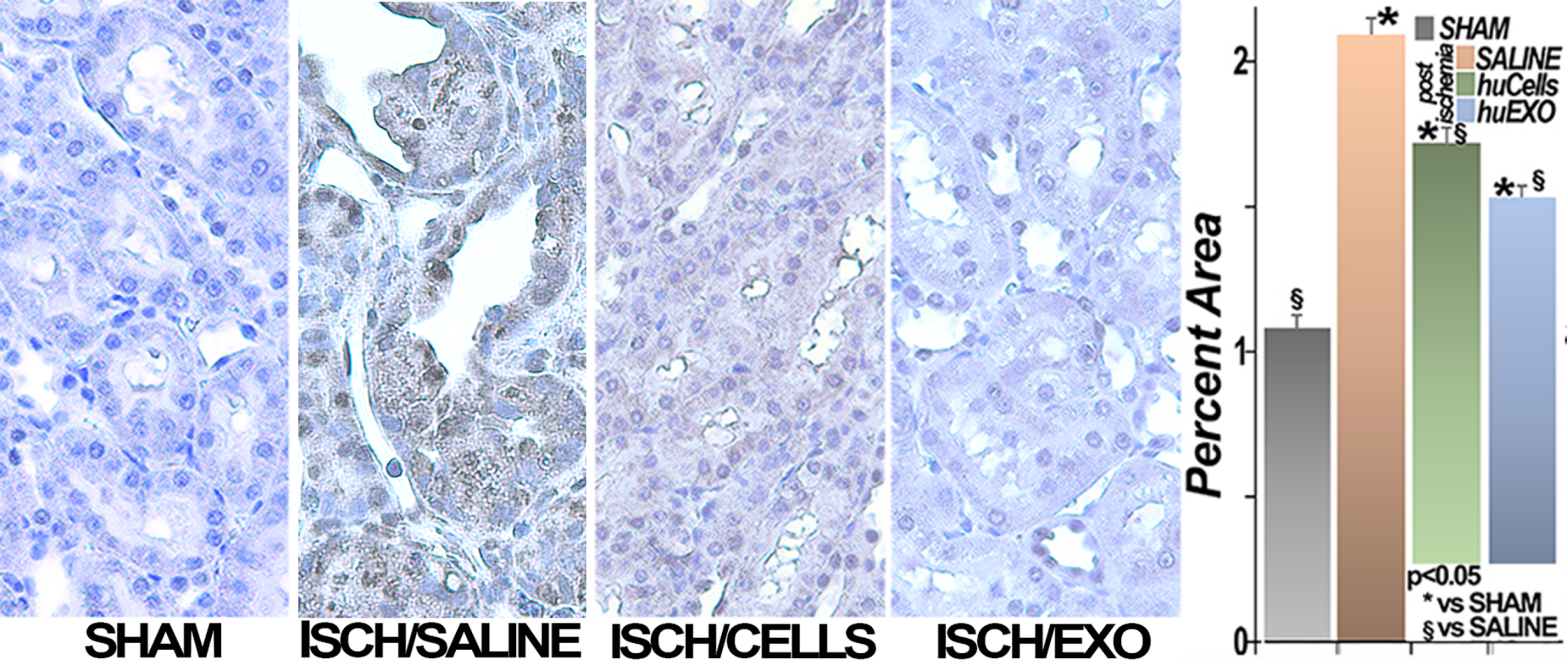
Oxidative stress. Representative images of renal 4-hydroxynonenal adducts (HNE) are shown. HNE adducts (brown) were seen in untreated ischemic (ISCH/SALINE) rats, and nearly undetectable in sham rats (SHAM). Treatment with renal cells (ISCH/CELLS) or their exosomes (ISCH/EXO) prevented HNE adduct formation. Nuclei are counterstained blue with hematoxylin. The bar diagram summarizes the results: (*), significantly higher than the sham rat group (SHAM), p < 0.05. (§), significantly lower than untreated ischemic rat group (SALINE), p < 0.05. (n = 77 measurements per group).

**Fig 10:**
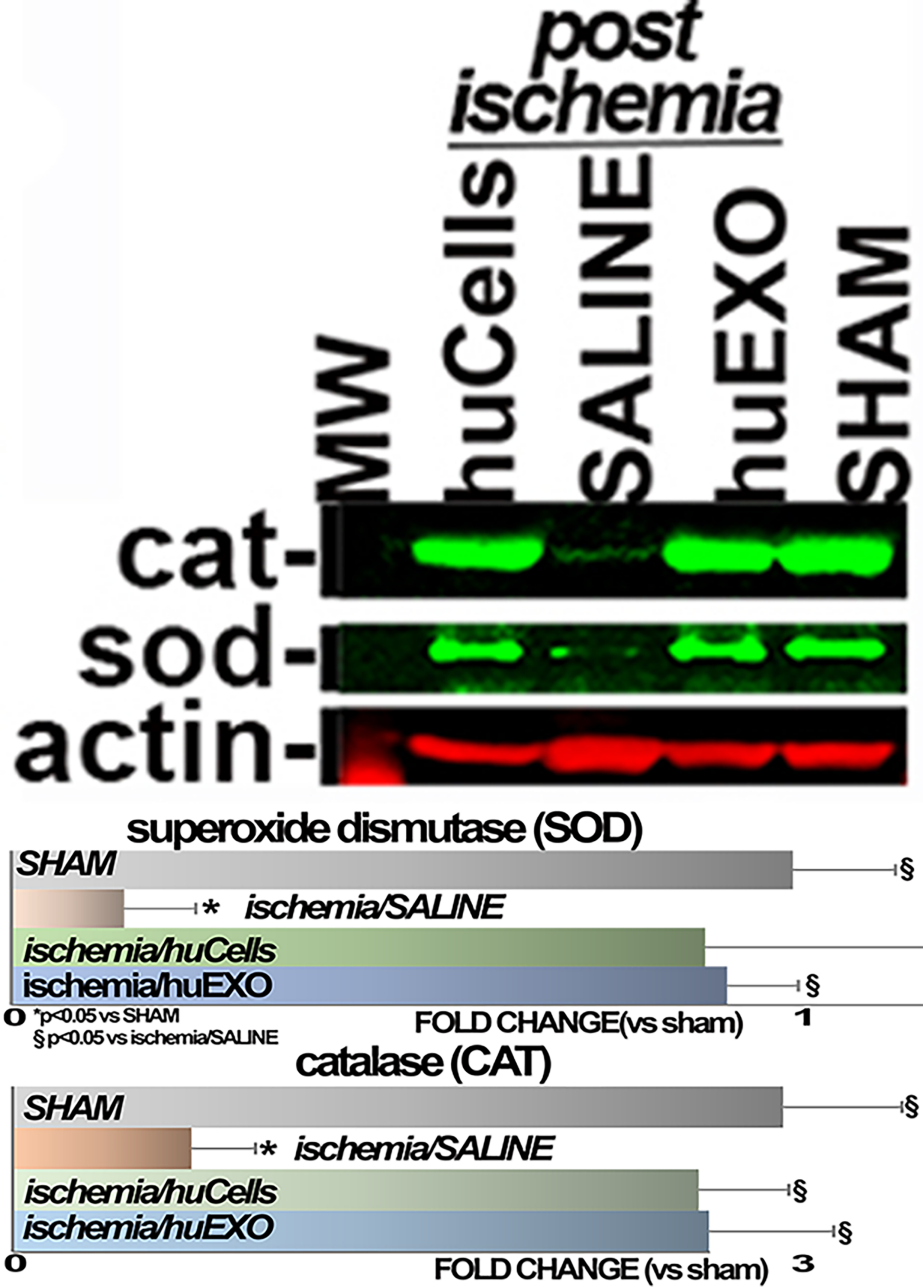
Anti-oxidant proteins. Top, specific redox related proteins, catalase (CAT), and superoxide dismutase (SOD), were measured on western blots (n = 4). Bottom, the horizontal bar diagram summarizes SOD and CAT protein levels. (*), significantly lower than sham rat group (SHAM), p < 0.05. (§), significantly higher than untreated ischemic group (SALINE), p < 0.05.

We also examined the preventive role of cell and exosome treatments on oxidant stress post-ischemia. For this purpose, renal sections were immune-stained for 4-hydroxynonelal (HNE) adducts, the footprints of oxidative stress [11]. In Fig 9 is shown that renal HNE adduct formation was undetectable in sham kidneys, but increased 2.1 fold in kidneys of untreated ischemic rats. Moreover, treatment with either human renal cells, or their exosomes, prevented HNE adduct formation. We also found that renal levels of catalase and superoxide dismutase were severely suppressed post-ischemia, whereas infusions of human renal cells, or their exosomes, protected the expression of both anti-oxidant enzymes, Fig 10.

### Stress proteins

**Fig 11:**
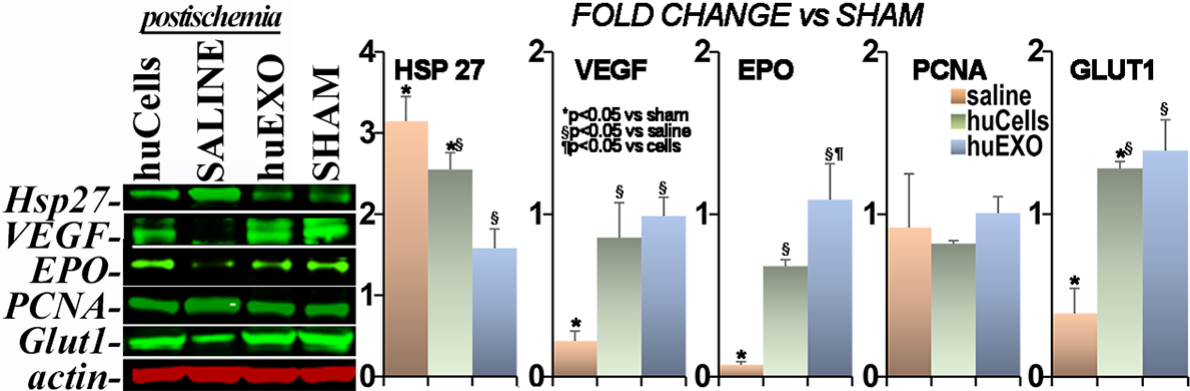
Stress-related proteins. Representative immunoblot of five proteins (top to bottom, n = 4): Heat Shock Protein 27 (HSP27) was activated in untreated ischemia group (SALINE) with respect to sham group (SHAM), (*) p < 0.05. HSP27 levels were partly normalized with cell infusions (huCells), or by exosomes (huEXO, (§), p <0.05. Vascular endothelial growth factor (VEGF) levels were suppressed in untreated ischemia compared to the sham group (*), p <0.05. However, VEGF levels in ischemia were normalized with cell infusions (huCells), or by exosomes (huEXO, (§), p <0.05. Erythropoietin (EPO) was suppressed in untreated ischemia with respect to the sham group (*), p <0.05. EPO levels improved with cell infusions (huCells), and nearly corrected by exosomes (huEXO), (§), p <0.05. In fact, renal exosomes appeared superior to their cells at correcting EPO levels post-ischemia, (¶), p < 0.05. Proliferating cell nuclear antigen (PCNA) was relatively constant in all experimental groups with respect to sham group. GLUT1 (Slc2a1) was suppressed by untreated ischemia with respect to the sham group, (*), p < 0.05, improved significantly in cell treated ischemia, (§), p <0.05, and was corrected with exosomes. Actin was included for normalization purposes.

The multiple pathogenic alterations in the post-ischemic kidney include significant changes in renal gene expression [11]. For instance, levels of the stress protein HSP27 were increased in untreated ischemic kidneys [11], and less so in the treated groups. In contrast, several protein members of the hypoxia-inducible factor α1 were suppressed in untreated ischemia, including vascular endothelial growth factor, erythropoietin, and glucose transporter 1 [26], while cell and exosome treatment limited the alterations, Fig 11.

### Fibrosis and inflammation transcripts

**Fig 12:**
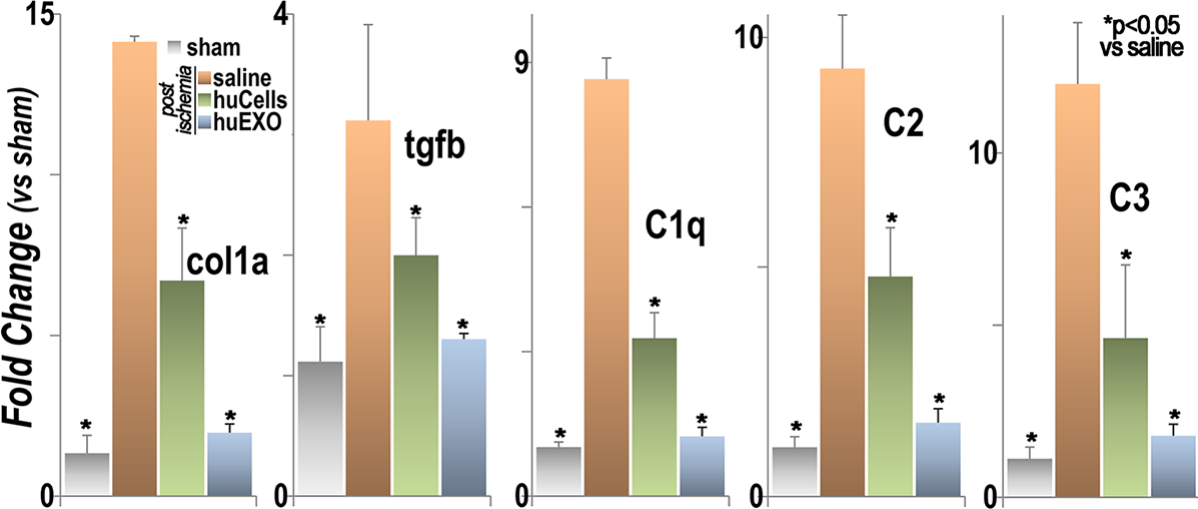
Two sets of renal transcripts were amplified by RT-PCR. Collagen type 1 alpha 1 chain (col1a) and transforming growth factor beta 1 (tgfb), representing a pro-fibrogenic genotype, were activated by untreated ischemia and partly corrected by infusions of cells or their exosomes (*), p < 0.05. Complement components C1q, C2, and C3, representing a pro-inflammatory genotype, were activated in untreated ischemia, and partly corrected by infusions of cells or their exosomes, (*) p < 0.05.

We examined several renal transcripts related to fibrosis and inflammation by RT-PCR, Fig 12. We found that collagen type 1 alpha 1 and transforming growth factor beta 1 were substantially increased in untreated ischemia. However, infusions of human kidney cells, or their exosomes, prevented the activation of the pro-fibrogenic genes. In addition, renal transcripts encoding complement components C1, C2 and C3 were activated in untreated renal ischemia, whereas treatment with human kidney cells, or their exosomes, prevented renal complement activation.

### Proteomic analysis post-ischemia and after cell and exosome therapy

**Fig 13:**
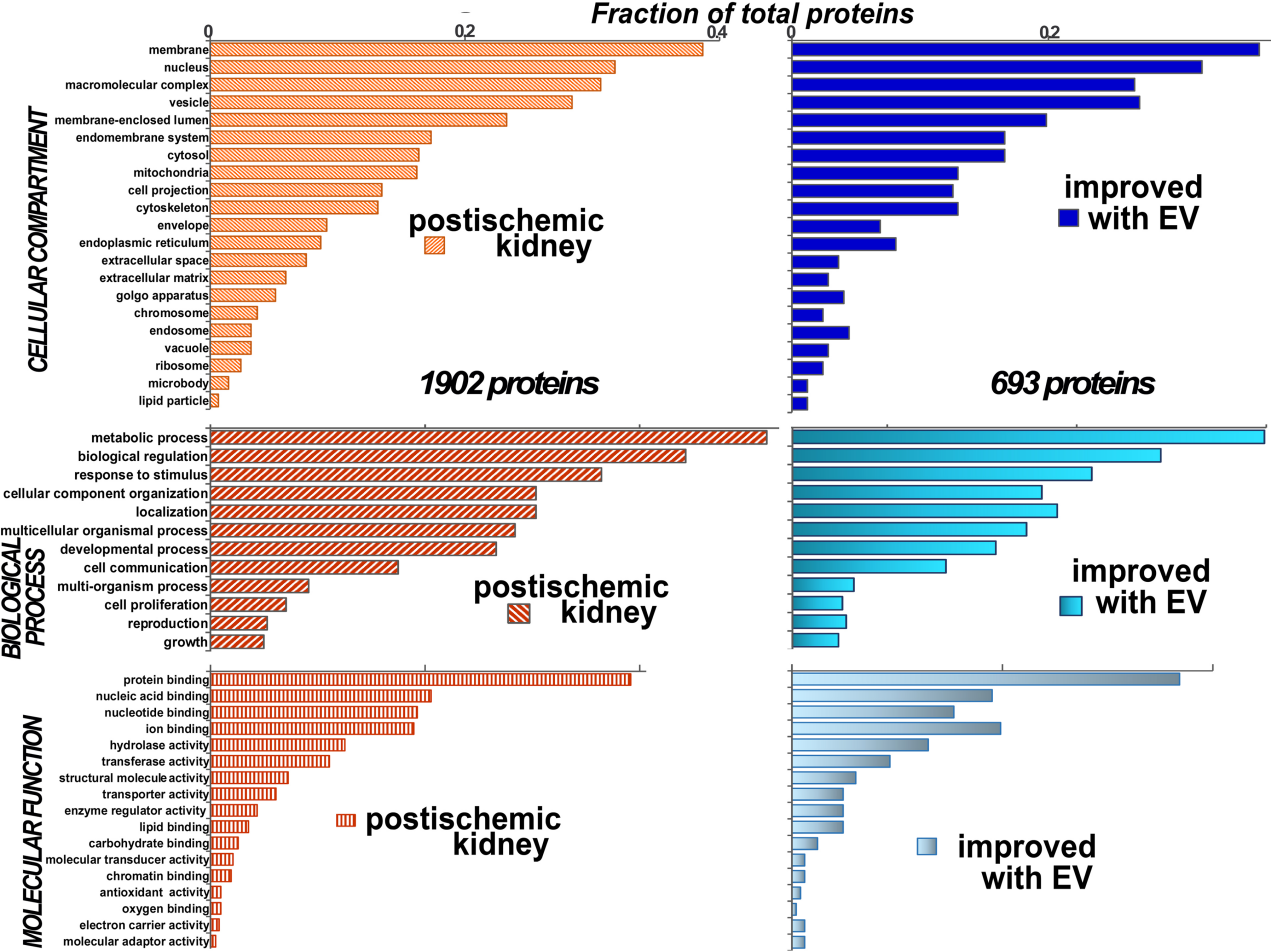
Distribution of proteins in post-ischemic rats untreated or treated with exosomes. There were 1902 proteins altered post-ischemia (left top, middle and lower orange panels). These proteins represented most cellular compartments (top left), biological processes (middle left) or molecular functions (lower left). There were 693 altered proteins that were improved with exosomes (right top, middle, and bottom blue panels). These proteins were partly, or completely, normalized by exosomes with respect to sham control rats, p < 0.05. The improved proteins were also from all cellular compartments (top), biological processes (middle), and molecular function (bottom). Some of the altered and improved proteins were assigned to more than one category.

Rat kidney proteins were analyzed by the IUSM Proteomics Core Facility, addressing two objectives. First, we identified and measured renal proteins. Second, we then tested the hypothesis that levels of affected renal proteins in post-ischemic kidneys were protected by infusion of cells and exosomes. We were able to identify and measure levels of 6150 renal proteins, representing all cell compartments, comprising most biological processes and molecular functions. Proteins in 1902 functional categories (Fig 13) were altered post-ischemia, and exosomes significantly improved the levels in 36%, p < 0.05. Improvement signifies a partial or complete preservation, by exosomes, of altered proteins levels in reference to untreated ischemic protein levels; whether by significantly limiting the upregulation of proteins levels or by limiting the suppression of inhibited proteins; Fig 13.

The number of sequenced proteins likely represents a portion of the entire proteome, if one considers that the number of rat renal transcripts is much larger [11]. In any event, 628 unique proteins were altered 6 days post-ischemia, and of these, 472 proteins were elevated and 156 depressed. In the post-ischemic group treated with exosomes, 280 upregulated proteins were significantly improved in part or in full, and 97 suppressed proteins were significantly improved in part of in full, S1 Table, supplement. The effects of cell infusions on renal protein alterations were more modest, S2 Table, supplement. It is noteworthy that the cellular distribution of altered proteins post-ischemia and of improved proteins by exosome infusions were similar, consistent with broad protection of protein expression post-ischemia.

### Exosomes and renal tubular cell viability

**Fig 14:**
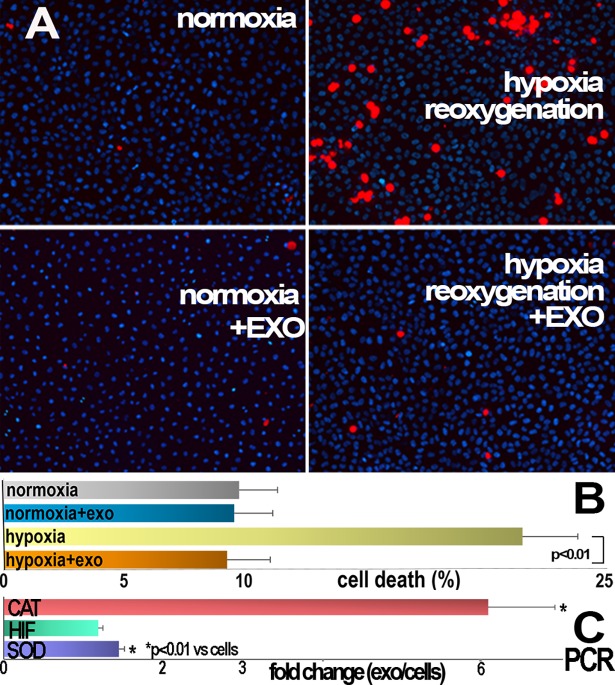
Rat renal tubular cell death by hypoxia/re-oxygenation and the role of human tubular cell exosomes. Primary renal tubular cells were divided into four groups and cultured on 35 mm dishes in normoxic medium (38% O_**2**_ and 5% CO_**2**_) for 5 days. Two groups were continued in normoxic medium for 36 more hours (left, normoxia). One of these groups received exosomes during the final 24 hours (not shown). The other two groups were switched to hypoxic medium (1% O_**2**_ and 5% CO_**2**_) for 12 hours and then one hypoxic group was returned to normoxic medium for 24 more hours (hypoxia). One hypoxic group was also returned to normoxic medium which contained added human renal cell exosomes (right, HYPOXIA/EXO). Cells were then incubated with propidium iodide, fixed and individual dead cells visualized (red dots) and counted. The bar diagram summarizes the average number of dead cells measured in the dishes (n = 5). The number of dead cells in hypoxia/re-oxygenation (hypoxia) was higher than in normoxia, and cell death was significantly limited by exosomes (hypoxia/EXO). The bar diagram on right summarizes the enrichment of catalase (CAT) and superoxide dismutase (SOD) transcripts in exosomes with respect to the originating cells and normalized for actin mRNA. Both anti-oxidant transcripts were significantly higher in exosomes than in the originating cells (n = 5).

We tested the protective role of human renal exosomes on the viability of cultured rat renal tubular cells subjected to hypoxia (1% O_**2**_ and 5% CO_**2**_) for 12 hours, as this could potentially represent an *in vitro* potency test to anticipate the beneficial biological effect. The exosomes were added to the culture medium during the 24-hour period of re-oxygenation (38% O_**2**_ and 5% CO_**2**_) that immediately followed hypoxia. Following hypoxia/re-oxygenation there was a noticeable and significant increase in cell death, which was substantially limited by exosome treatment. The addition of exosomes to normoxic cells did not alter cell death (0.94 ± 0.02 fold vs normoxic cells, p = NS). The human renal exosomes were significantly enriched, over the originating cells, in catalase and superoxide dismutase transcripts, which have the potential to afford protection during re-oxygenation stress, Fig 14. Exosomes from previously hypoxic rat tubular cells (HPC exo) were more protective than exosomes derived from normoxic cells (Table 1). This was not the case for exosome derived from human tubular cells, perhaps due to the ischemia during kidney harvest.

**Table 1:** Cell death in rat cells exposed to normoxic and previously hypoxic (preconditioned) rat exosomes.

			p value		
cells	exosomes	cell death (%)	v normoxia	v hypoxia/no exo	vs hypoxia/normoxic exo
normoxia	none	2.4 ± 0.6	—	0.002	0.07
hypoxia	none	12.4 ± 2.7	0.002	—	0.01
hypoxia	normoxic	4.4 ± 0.9	0.07	0.01	—
hypoxia	hypoxic	1.0 ± 0.3	0.07	0.0008	0.002

## Discussion

We tested the hypothesis that intravenous administration of normal human kidney tubular cells, or their exosomes, prevented damage after hypoxic AKI. This goal was prompted by reports that stem cells and their exosomes improve renal structure in mice with glycerol-induced AKI [27, 28], and by our recent work with rat renal exosomes [11]. Accordingly, we presupposed that human renal exosomes, released ex vivo by donor cells, could be protective in hypoxic AKI. We compared the effects of human kidney tubular cells, or their exosomes, on Nude (RNU) rats with hypoxic AKI. We also wanted to compare the potential of human kidney cells, or their exosomes, in the prevention of irreversible hypoxic AKI damage and eventual chronic kidney disease (CKD). This latter goal was conceived as a potential alternative to kidney transplantation, which is not keeping up with the needs of expecting recipients [29]. Moreover, up to 17% of recovered kidneys are discarded [30], and exosomes from the discarded kidneys might become an untapped therapeutic resource.

We studied four groups of Nude rats: sham, untreated renal ischemia, ischemia treated with intravenous human renal tubular cells, and ischemia treated with intravenous exosomes harvested from the same cells during three days prior to transplantation. The cells and exosomes were subjected to hypoxic preconditioning as that can provide added protection over normoxia [11], although the human kidneys had already been subjected to ischemia during harvest. We evaluated differences in serum creatinine and renal changes that contribute to chronicity: microvascular disruption and loss, tubular cell death, inflammation, oxidative stress and fibrosis [11]. We also examined the renal proteome post-ischemia and the effects of cell and exosome infusions on renal protein expression. We employed Nude rats, which lack mature T cells, and do not reject transplanted tissue from other species [31], although are still able to mount an immune response to ischemic renal injury [32]. Nevertheless, extrapolating our data to immune competent animals must be qualified in this context. Undeniably, ischemic rats had profound functional and structural alterations at 6 days of renal reperfusion: There was a rapid rise in serum creatinine that peaked at 48 hours of reperfusion, followed by a slow and partial correction. In addition, untreated renal ischemia caused organomegaly and renal fibrosis (below). Tubular damage and apoptosis were extensive, and renal neutrophils intermixed with broadly distributed interstitial fibrosis. There was also widespread loss of the renal microvasculature and generalized tubular HNE adduct formation, a foot print of oxidative stress. These severe renal changes of advanced ischemic renal injury [11] were largely prevented by intravenous infusions of exosomes and to a lesser extent by cell infusions.

HLA-A1 from human donor exosomes or cells was found in host tubules, possibly a site of action. Potentially, the vast numbers of injected exosomes, 13.6E10 exosomes/rat, could reach a maximum of 2.12E06 exosomes/nephron, assuming 64,000 recipient nephrons per 2 kidneys [33], although this does not rule out vascular localization. The same calculation for 3E06 injected kidney cells yields a maximum of 47 cells reaching each nephron, where they appear to localize alongside host tubular cells.

We presume that the main action of exosomes, and perhaps of cells, was functional restoration, and base this idea on the rapid recovery after injections. We infer that kidneys were in a dysfunctional state during the initial 24 hours of reperfusion, but still viable and capable of nearly full recovery following treatments [11]. This interpretation also accounts for the lack of significant chronicity in the treated ischemic groups, compared to the untreated group. The proteome data further support a rapid functional correction at 24 hours, in that while the renal proteome was extensively altered by ischemia, exosomes limited proteomic alterations. The most logical interpretation of these acute and broad effects is that exosomes, directly or indirectly, improved blood flow and delivery of nutrients and oxygen to affected cells [11]. On the other hand, human renal exosomes might directly limit tubular death after re-oxygenation, as shown in vitro. This action might be mediated in part by contributing to the anti-oxidant potential of injured cells. Ultimately, further studies are necessary to answer these questions.

## Supporting information

S1 Table Proteins altered with ischemia and improved with exosome treatment. (DOCX)

S2 Table Proteins altered with ischemia and improved with cell treatment. (DOCX)

S1 Appendix Methods. Description of experimental techniques (methods).
(DOCX)
