# Supplementary material for: Human extracellular microvesicles from renal tubules reverse kidney ischemia-reperfusion injury in rats
Source: PLoS One. 2018 Aug 27;13(8):e0202550. doi: 10.1371/journal.pone.0202550 (PMC6110463; doi:10.1371/journal.pone.0202550)
Supplement: S1 Table — (DOCX) [file pone.0202550.s001.docx]

| Table 1S | |
| --- | --- |
| PROTEINS PROTECTED BY EXOSOMES FROM SUPPRESSION IN ISCHEMIA (GRAY)– *OR FROM ACTIVATION IN ISCHEMIA (BLUE)* | |
| *G3V6P7* | *Myosin, heavy polypeptide 9, non-muscle, Myh9* |
| P15999 | ATP synthase subunit alpha, mitochondrial, Atp5a1 |
| *P34058* | *Heat shock protein HSP 90-beta, Hsp90ab1* |
| P63039 | 60 kDa heat shock protein, mitochondrial*,* Hspd1 |
| *G3V852* | *Protein Tln1* |
| G3V7J0 | Aldehyde dehydrogenase family 6, subfamily A1, isoform CRA_b, Aldh6a1 |
| *M0R9X8* | *Cytoplasmic dynein 1 heavy chain 1, Dync1h1* |
| *C0JPT7* | *Filamin alpha, Flna* |
| *A0A0G2JUA5* | *Protein Ahnak* |
| A0A0G2JTL5 | Pyruvate carboxylase, mitochondrial, Pc |
| Q9ER34 | Aconitate hydratase, mitochondrial, Aco |
| P09034 | Argininosuccinate synthase, Ass1 |
| P31977 | Ezrin, Ezr |
| *A0A0A0MY09* | *Endoplasmin, Hsp90b1* |
| *A0A0G2K8V2* | *Vinculin, Vcl* |
| *P06761* | *78 kDa glucose-regulated protein, Hspa5* |
| P19112 | Fructose-1,6-bisphosphatase 1, Fbp1 |
| P04636 | Malate dehydrogenase, mitochondrial, Mdh2 |
| P56574 | Isocitrate dehydrogenase [NADP], mitochondrial ,Idh2 |
| P02761 | Major urinary protein |
| O88989 | Malate dehydrogenase, cytoplasmic, Mdh1 |
| *P05197* | *Elongation factor 2, Eef2* |
| *P18418* | *Calreticulin , Calr* |
| P10860 | Glutamate dehydrogenase 1, mitochondrial, Glud1 |
| *A0A0H2UHM5* | *Protein disulfide-isomerase, Pdia3* |
| Q64428 | Trifunctional enzyme subunit alpha, mitochondrial, Hadha |
| B2GV06 | Succinyl-CoA:3-ketoacid coenzyme A transferase 1, mitochondrial, Oxct1 |
| *Q6S3A0* | *Plectin, Plec* |
| P17764 | Acetyl-CoA acetyltransferase, mitochondrial, Acat1 |
| Q9JJ40 | Na(+)/H(+) exchange regulatory cofactor NHE-RF3, Pdzk1 |
| P11517 | Hemoglobin subunit beta-2 |
| G3V6S2 | Aconitate hydratase, Aco1 |
| *A0A0G2JXT8* | *Protein Flnb* |
| A0A0G2JSN9 | Kidney-specific protein (KS), isoform CRA_a, Acsm2a |
| *F1LNF1* | *Heterogeneous nuclear ribonucleoproteins A2/B1, Hnrnpa2b1* |
| *Q63610* | *Tropomyosin alpha-3 chain, Tpm3* |
| D4A133 | Protein Atp6v1a |
| *A0A0G2JZ52* | *Protein Hnrnpu* |
| P27867 | Sorbitol dehydrogenase, Sord |
| G3V6S5 | C-1-tetrahydrofolate synthase, cytoplasmic, Mthfd1 |
| *D3ZLY9* | *Histone H2B, Hist1h2bh* |
| *F1M853* | *Protein Rrbp1* |
| D3ZXY4 | Protein Aldh8a1 |
| P85834 | Elongation factor Tu, mitochondrial, Tufm |
| *P08932* | *T-kininogen 2* |
| A0A0G2JSI1 | 4-trimethylaminobutyraldehyde dehydrogenase, Aldh9a1 |
| Q9WVK7 | Hydroxyacyl-coenzyme A dehydrogenase, mitochondrial, Hadh |
| P00502 | Glutathione S-transferase alpha-1, Gsta1 |
| *Q6P3V8* | *Eukaryotic translation initiation factor 4A1, Eif4a1* |
| Q66HF1 | NADH-ubiquinone oxidoreductase 75 kDa subunit, mitochondrial, Ndufs1 |
| A0A0G2JSV6 | Protein Hba-a2 |
| *Q5XFX0* | *Transgelin-2, Tagln2* |
| A0A0G2JVM0 | Delta-1-pyrroline-5-carboxylate dehydrogenase, mitochondrial, Aldh4a1 |
| P14408 | Fumarate hydratase, mitochondrial, Fh |
| Q60587 | Trifunctional enzyme subunit beta, mitochondrial, Hadhb |
| *A0A0G2K9I6* | *Ceruloplasmin, Cp* |
| *Q5U328* | *Nucleolin, Ncl* |
| P07379 | Phosphoenolpyruvate carboxykinase, cytosolic [GTP], Pck1 |
| G3V6P2 | Dihydrolipoamide S-succinyltransferase, isoform CRA_a, Dlst |
| *P07150* | *Annexin A1, Anxa1* |
| Q68FT3 | Pyridine nucleotide-disulfide oxidoreductase domain-containing protein 2, Pyroxd2 |
| *P09495* | *Tropomyosin alpha-4 chain, Tpm4* |
| Q8CG45 | Aflatoxin B1 aldehyde reductase member 2, Akr7a2 |
| A0A0H2UHE1 | Succinate--CoA ligase [ADP/GDP-forming] subunit alpha, mitochondrial, Suclg1 |
| *F1MAA7* | *Protein Lamc1* |
| *A0A0G2KAJ7* | *Collagen alpha-1(XII) chain, Col12a1* |
| *P62963* | *Profilin-1, Pfn1* |
| *P24368* | *Peptidyl-prolyl cis-trans isomerase B, Ppib* |
| *A0A096P6L8* | *Fibronectin, Fn1* |
| P62815 | V-type proton ATPase subunit B, brain isoform, Atp6v1b2 |
| *Q5D059* | *Heterogeneous nuclear ribonucleoprotein K, Hnrnpk* |
| *Q6URK4* | *Heterogeneous nuclear ribonucleoprotein A3, Hnrnpa3* |
| *Q5RJR9* | *Serine (Or cysteine) proteinase inhibitor, clade H, member 1, isoform CRA_b, Serpinh1* |
| *G3V9M6* | *Fibrillin 1, isoform CRA_a, Fbn1* |
| P24329 | Thiosulfate sulfurtransferase, Tst |
| *P63245* | *Receptor of activated protein C kinase 1, Rack1* |
| *A0A0G2JWK7* | *Transgelin, Tagln* |
| *P49242* | *40S ribosomal protein S3a, Rps3a* |
| P04904 | Glutathione S-transferase alpha-3, Gsta3 |
| *G3V786* | *Protein Akr1b10* |
| *A0A0G2JZI2* | *Protein Eprs* |
| D3ZCI0 | Glycerol kinase, Gk |
| *G3V7U4* | *Lamin-B1, Lmnb1* |
| G3V7L8 | ATPase, H+ transporting, V1 subunit E isoform 1, isoform CRA_a, Atp6v1e1 |
| *D3ZQN7* | *Protein Lamb1* |
| *P62909* | *40S ribosomal protein S3, Rps3* |
| *F1MAN8* | *Laminin, alpha 5, isoform CRA_a, Lama5* |
| *G3V699* | *Protein transport protein Sec31A* |
| F1LP30 | Methylcrotonoyl-CoA carboxylase subunit alpha, mitochondrial, Mccc1 |
| *D3ZUL3* | *Protein Col6a1* |
| Q5U2Q3 | Ester hydrolase C11orf54, |
| *P19945* | *60S acidic ribosomal protein P0, Rplp0* |
| A0A0G2JSV5 | 3-hydroxyanthranilate 3,4-dioxygenase, Haao |
| Q5XIT9 | Methylcrotonoyl-CoA carboxylase beta chain, mitochondrial, Mccc2 |
| Q6AYS3 | Carboxypeptidase, Ctsa |
| B2GV15 | Dihydrolipoamide acetyltransferase component of pyruvate dehydrogenase complex, Dbt |
| P56571 | ES1 protein homolog, mitochondrial, |
| *Q6T487* | *Alpha-actinin-1, Actn1* |
| *G3V9E3* | *Caldesmon 1, isoform CRA_b, Cald1* |
| *A0A0G2K6J5* | *Myosin light polypeptide 6, Myl6* |
| A0A0G2JSW3 | Hemoglobin subunit beta-1, Hbb |
| Q63342 | Dimethylglycine dehydrogenase, mitochondrial, Dmgdh |
| *P07335* | *Creatine kinase B-type, Ckb* |
| A2VCW9 | Alpha-aminoadipic semialdehyde synthase, mitochondrial, Aass |
| *A0A0H2UHX3* | *40S ribosomal protein S4, Rps4x* |
| Q4KLP0 | Probable 2-oxoglutarate dehydrogenase E1 component DHKTD1, mitochondrial, Dhtkd1 |
| *P85968* | *6-phosphogluconate dehydrogenase, decarboxylating, Pgd* |
| P21775 | 3-ketoacyl-CoA thiolase A, peroxisomal, Acaa1a |
| P04041 | Glutathione peroxidase 1, Gpx1 |
| P50554 | 4-aminobutyrate aminotransferase, mitochondrial, Abat |
| *Q5XI73* | *Rho GDP-dissociation inhibitor 1, Arhgdia* |
| *A0A0G2JSW0* | *Myosin regulatory light chain 12B, Myl12b* |
| *P47942* | *Dihydropyrimidinase-related protein 2, Dpysl2* |
| *Q6AYI1* | *DEAD (Asp-Glu-Ala-Asp) box polypeptide 5, Ddx5* |
| Q63150 | Dihydropyrimidinase, Dpys |
| P23764 | Glutathione peroxidase 3, Gpx3 |
| *Q5FVM4* | *Non-POU domain-containing octamer-binding protein, Nono* |
| Q6AYQ8 | Acylpyruvase FAHD1, mitochondrial, Fahd1 |
| Q02974 | Ketohexokinase, Khk |
| A0A0G2JSS8 | Peroxiredoxin 5, isoform CRA_c, Prdx5 |
| F1LRY5 | RCG45398, Sardh |
| *D4A9L2* | *Protein Srsf1* |
| *F1LNH3* | *Procollagen, type VI, alpha 2, isoform CRA_a, Col6a2* |
| O08651 | D-3-phosphoglycerate dehydrogenase, Phgdh |
| A0A0G2K9J2 | Protein Atp6v1h |
| *Q3KRF2* | *High density lipoprotein binding protein (Vigilin), Hdlbp* |
| *Q63798* | *Proteasome activator complex subunit 2, Psme2* |
| *Q3MIE4* | *Synaptic vesicle membrane protein VAT-1 homolog, Vat1* |
| Q62669 | Protein Hbb-b1 |
| *D3ZU13* | *Protein Eif4g1* |
| P97532 | 3-mercaptopyruvate sulfurtransferase, Mpst |
| *P68511* | *14-3-3 protein eta, Ywhah* |
| A0A0A0MY00 | Short/branched chain-specific acyl-CoA dehydrogenase, mitochondrial, Acadsb |
| *Q6P736* | *Polypyrimidine tract binding protein 1, isoform CRA_a, Ptbp1* |
| *A0A0G2K014* | *Protein Lcp1* |
| F1LZL1 | Sulfotransferase |
| *A0A0G2JSZ5* | *Protein disulfide-isomerase A6, Pdia6* |
| P29266 | 3-hydroxyisobutyrate dehydrogenase, mitochondrial, Hibadh |
| *P02454* | *Collagen alpha-1(I) chain, Col1a1* |
| *P14669* | *Annexin A3, Anxa3* |
| *D3ZVQ0* | *Protein Usp5* |
| *Q6PDW1* | *40S ribosomal protein S12, Rps12* |
| *F1LNJ2* | *U5 small nuclear ribonucleoprotein 200 kDa helicase, Snrnp200* |
| *F1LR02* | *Procollagen, type XVIII, alpha 1, isoform CRA_a, Col18a1* |
| *A0A0G2JWC7* | *Protein Fermt2* |
| *P62329* | *Thymosin beta-4, Tmsb4x* |
| *P0DMW1* | *Heat shock 70 kDa protein 1B, Hspa1b* |
| *I6L9G6* | *Protein Tardbp* |
| Q8R5M5 | 2-amino-3-carboxymuconate-6-semialdehyde decarboxylase, Acmsd |
| *Q91ZN1* | *Coronin-1A, Coro1a* |
| *D3ZH41* | *Cytoskeleton-associated protein 4 (Predicted), Ckap4* |
| *P62914* | *60S ribosomal protein L11, Rpl11* |
| *P62718* | *60S ribosomal protein L18a, Rpl18a* |
| *Q63413* | *Spliceosome RNA helicase Ddx39b, Ddx39b* |
| *A0A0G2K8H0* | *Caprin-1* |
| D3ZPV8 | Protein Ggct |
| *Q80Z29* | *Nicotinamide phosphoribosyltransferase, Nampt* |
| *P11762* | *Galectin-1, Lgals1* |
| *Q4AEF8* | *Coatomer subunit gamma-1, Copg1* |
| *E9PT66* | *Protein Sf3b3* |
| *Q8VHK7* | *Hepatoma-derived growth factor, Hdgf* |
| *A0A0G2JTG7* | *Heterogeneous nuclear ribonucleoprotein H, Hnrnph1* |
| *Q5U2S7* | *Proteasome (Prosome, macropain) 26S subunit, non-ATPase, 3, Psmd3* |
| *Q794E4* | *Heterogeneous nuclear ribonucleoprotein F, Hnrnpf* |
| Q66HG4 | Aldose 1-epimerase, Galm |
| Q7TQ94 | Nitrilase homolog 1, Nit1 |
| *P41542* | *General vesicular transport factor p115, Uso1* |
| *F2Z3Q8* | *Importin subunit beta-1, Kpnb1* |
| *Q5RJR8* | *Leucine-rich repeat-containing protein 59, Lrrc59* |
| *G3V6S8* | *Serine/arginine-rich splicing factor 6, Srsf6* |
| *D4A9D6* | *DEAH (Asp-Glu-Ala-His) box polypeptide 9 (Predicted), Dhx9* |
| *P47860* | *ATP-dependent 6-phosphofructokinase, platelet type, Pfkp* |
| Q5PQZ9 | NADH dehydrogenase [ubiquinone] 1 subunit C2, Ndufc2 |
| *P52925* | *High mobility group protein B2, Hmgb2* |
| *P01048* | *T-kininogen 1, Map1* |
| *P63086* | *Mitogen-activated protein kinase 1, Mapk1* |
| *P51886* | *Lumican, Lum* |
| Q5I0P2 | Glycine cleavage system H protein, mitochondrial, Gcsh |
| *P63326* | *40S ribosomal protein S10, Rps10* |
| *G3V6H2* | *Pre-mRNA processing factor 8, isoform CRA_a, Prpf8* |
| *G3V8R0* | *Protein RGD1311703* |
| *P48675* | *Desmin, Des* |
| *P17077* | *60S ribosomal protein L9, Rpl9* |
| *D4A781* | *Protein Ipo5* |
| *P69897* | *Tubulin beta-5 chain, Tubb5* |
| *Q0ZFS8* | *Protein Srsf3* |
| Q0D2L3 | Agmatinase, mitochondrial, Agmat |
| *Q64598* | *Histone H2A type 1-F* |
| A0A0G2K6A9 | Protein RUFY3 |
| *Q4KMA8* | *Endoplasmic reticulum aminopeptidase 1, Erap1* |
| *G3V8T5* | *RuvB-like helicase, Ruvbl2* |
| G3V8U8 | Branched-chain-amino-acid aminotransferase, Bcat2 |
| *Q6AXS5* | *Plasminogen activator inhibitor 1 RNA-binding protein, Serbp1* |
| *Q63945* | *Protein SET,* |
| A0A0G2JSX3 | Na+ dependent glucose transporter 1, isoform CRA_b, Naglt1 |
| *B2RYG5* | *Protein Taf15* |
| *B1WC49* | *Api5 protein, Api5* |
| *D3ZXP3* | *Histone H2A, H2afx* |
| *A0A096MIX2* | *DEAD (Asp-Glu-Ala-Asp) box polypeptide 17, isoform CRA_a, Ddx17* |
| *A0A0G2K121* | *Malectin, Mlec* |
| *G3V9T9* | *Tyrosine-protein phosphatase non-receptor type, Ptpn6* |
| *Q66H61* | *Glutaminyl-tRNA synthetase, Qars* |
| Q4QQW3 | Hydroxyacid-oxoacid transhydrogenase, mitochondrial, Adhfe1 |
| *A0A0G2JW60* | *Protein Utrn, Utrn* |
| *D3Z9E1* | *Elastin microfibril interfacer 1 (Predicted), isoform CRA_b, Emilin1* |
| *Q6DGF2* | *Enthoprotin, Clint1* |
| *A0A0G2K9V6* | *Threonine--tRNA ligase, cytoplasmic, Tars* |
| *Q5RKH2* | *Galactokinase 1, Galk1* |
| *Q62952* | *Dihydropyrimidinase-related protein 3, Dpysl3* |
| *Q80U96* | *Exportin-1, Xpo1* |
| *Q9QZR6* | *Septin-9, Sept9* |
| *Q66HC5* | *Nuclear pore complex protein Nup93* |
| *Q05982* | *Nucleoside diphosphate kinase A, Nme1* |
| *F1LM55* | *Protein Ccar2* |
| *Q5U2U3* | *Poly [ADP-ribose] polymerase, Parp3* |
| *D3ZUB0* | *Protein Rcn1* |
| *G3V9D8* | *Carboxylic ester hydrolase* |
| Q9JJI3 | Alpha-2u globulin |
| P63095 | Guanine nucleotide-binding protein G(s) subunit alpha isoforms short, Gnas |
| *F1M265* | *Palladin, Palld* |
| *D3ZZC1* | *Protein Txndc5* |
| *P84083* | *ADP-ribosylation factor 5, Arf5* |
| *F1LMW7* | *Myristoylated alanine-rich C-kinase substrate, Marcks* |
| *D4A3K5* | *Histone H1.1, Hist1h1a* |
| *Q9WVH8* | *Fibulin-5, Fbln5* |
| *Q921A4* | *Cytoglobin, Cygb* |
| *E9PST5* | *Protein Acin1* |
| *Q2LAP6* | *Testin* |
| *Q6AYC4* | *Macrophage-capping protein, Capg* |
| *B1WC67* | *Protein Slc25a24* |
| *D4A5T8* | *Periplakin (Predicted), Ppl* |
| D4A197 | Methylmalonyl CoA epimerase (Predicted), isoform CRA_d, Mcee |
| *Q5RJK5* | *Chromobox homolog 3 (HP1 gamma homolog, Drosophila), Cbx3* |
| *A0A0G2K7X7* | *Oxidation resistance protein 1, C7* |
| *F1M9B2* | *Insulin-like growth factor binding protein 7, isoform CRA_b, Igfbp7* |
| *O08699* | *15-hydroxyprostaglandin dehydrogenase [NAD(+)], Hpgd* |
| *A0A0G2K9C0* | *Protein Vasp* |
| *A0A0H2UHZ0* | *Sodium-dependent phosphate transport protein 2B, Slc34a2* |
| *Q6B345* | *Protein S100-A11* |
| *A0A0G2K5N6* | *Rho-associated protein kinase, Rock2* |
| *Q62658* | *Peptidyl-prolyl cis-trans isomerase FKBP1A* |
| *G3V8Q1* | *Coatomer protein complex, subunit epsilon (Predicted), isoform CRA_c, Cope* |
| Q6PDU7 | ATP synthase subunit g, mitochondrial, Atp5l |
| *Q6IUR5* | *Neudesin, Nenf* |
| *D4A206* | *Protein Tcof1* |
| *P55009* | *Allograft inflammatory factor 1, Aif1* |
| *A0A0G2JXN6* | *Galectin, Lgals3* |
| *B5DFD8* | *SH3 domain-binding glutamic acid-rich-like protein, Sh3bgrl* |
| *O35346* | *Focal adhesion kinase 1, Ptk2* |
| *A0A096P6L9* | *Complement C5* |
| P08424 | Renin, Ren1 |
| *E9PTI6* | *Protein Raly* |
| *Q4QQT3* | *CUGBP Elav-like family member 1, Celf1* |
| Q9EPT8 | Chloride intracellular channel protein 5, Clic5 |
| *F1LWE6* | *Protein Msi2,* |
| *D4A4L4* | *Protein Map7d2* |
| *G3V763* | *Collagen alpha-1(V) chain, Col5a1* |
| *Q3SWS8* | *mRNA export factor, Rae1* |
| *F1LMA7* | *C-type mannose receptor 2, Mrc2* |
| F8WFT7 | Anion exchange protein, Slc4a1 |
| *P20595* | *Guanylate cyclase soluble subunit beta-1, Gucy1b3* |
| *B1WC16* | *BCL2-associated transcription factor 1, isoform CRA_a, Bclaf1* |
| *D3ZIY3* | *Protein Ythdf3* |
| *A0A0H2UHG3* | *Cullin 5, Cul5* |
| *P06302* | *Prothymosin alpha, Ptma* |
| *P29534* | *Vascular cell adhesion protein 1, Vcam1* |
| *B2RYC9* | *Glucosylceramidase, Gba* |
| *Q8CHN6* | *Sphingosine-1-phosphate lyase 1, Sgpl1* |
| *Q64361* | *Latexin, Lxn* |
| *A0A0G2K4T7* | *General transcription factor II-I, Gtf2i* |
| *Q3MHS7* | *GDP-mannose 4, 6-dehydratase, Gmds* |
| Q63910 | Alpha globin, Hba-a3 |
| *P62839* | *Ubiquitin-conjugating enzyme E2 D2, Ube2d2* |
| *A9CMB8* | *DNA helicase, Mcm6* |
| *A0A0G2K022* | *Protein Rcn3* |
| *G3V790* | *Transcription activator BRG1, Smarca4* |
| *Q5U2Z4* | *Nuclear factor of kappa light polypeptide gene enhancer in B-cells 2, p49/p100, Nfkb2* |
| *B1WC56* | *Nola2 protein, Nhp2* |
| *P13832* | *Myosin regulatory light chain RLC-A, Rlc-a* |
| *D3ZD83* | *Protein Mfsd10* |
| *Q497A2* | *D-serine modulator-1, Slc35b2* |
| *D4A2H2* | *Protein Sptlc1* |
| *G3V6S1* | *PRKC apoptosis WT1 regulator protein, Pawr* |
| *D4A5S9* | *Protein Prpf39* |
| *A0A0G2KAM4* | *Fascin, Fscn1* |
| Q4V897 | Coiled-coil domain-containing protein 90B, mitochondrial, Ccdc90b |
| *F1LRL9* | *Microtubule-associated protein 1B, Map1b* |
| *D3ZIV3* | *Protein Mad1l1* |
| *F1LQ00* | *Protein Col5a2* |
| *P16975* | *SPARC* |
| *Q3T1G7* | *Conserved oligomeric Golgi complex subunit 7, Cog7* |
| *P62142* | *Serine/threonine-protein phosphatase PP1-beta catalytic subunit, Ppp1cb* |
| *Q5PQN7* | *Protein LZIC* |
| *Q68FR8* | *Tubulin alpha-3 chain, Tuba3a* |
| *P81827* | *Urinary protein 1* |
| *A0A0G2K0M8* | *Neural cell adhesion molecule 1, Ncam1* |
| *A0A0G2JUB0* | *Protein FAM3C* |
| *Q9ERL1* | *Cytochrome b-245, beta polypeptide, isoform CRA_a, Cybb* |
| *F1LTU5* | *Protein NEWGENE_1311061, Zc3h6* |
| *Q4QQV0* | *Tubulin beta chain, Tubb6* |
| *Q09128* | *1,25-dihydroxyvitamin D(3) 24-hydroxylase, mitochondrial, Cyp24a1* |
| *P13233* | *2',3'-cyclic-nucleotide 3'-phosphodiesterase, Cnp* |
| Q7TP58 | Phosphoglycerate mutase, Bpgm |
| *A0A0G2K2M9* | *Protein Srrm,* |
| *D4ACF2* | *Protein Trim21* |
| *G3V6Y6* | *Alpha-1,4 glucan phosphorylase, Pygb* |
| *F1LQT0* | *DnaJ homolog subfamily C member 25, Dnajc25* |
| *D3ZT52* | *Protein Pbrm1* |
| M0R7V5 | Protein Tnfaip3 |
| *B0BNM9* | *Glycolipid transfer protein, GLTP* |
| *Q5BJP2* | *Spliceosome-associated protein CWC15 homolog, Cwc15* |
| *F1M5V2* | *Protein Glipr2* |
| *Q9QZQ5* | *Protein NOV homolog, Nov* |
| *Q62698* | *Cytoplasmic dynein 1 light intermediate chain 2, Dync1li2* |
| F1LNH7 | Sodium/nucleoside cotransporter, Slc28a1 |
| *P59924* | *THO complex subunit 1, Thoc1* |
| *A0A0G2K386* | *Protein Parn* |
| *F1M2Q1* | *Protein Arhgap42* |
| *D3ZFP4* | *DNA helicase, Mcm3* |
| *O70513* | *Galectin-3-binding protein, Lgals3bp* |
| *B0BNG2* | *Transmembrane 6 superfamily member 2, Tm6sf2* |
| A0A0G2JX00 | Kallikrein-1, Klk1c9 |
| *Q5U216* | *ATP-dependent RNA helicase DDX39A, Ddx39a* |
| *D4ADE5* | *Histone-lysine N-methyltransferase SETD7, Setd7* |
| *A0A0G2KB92* | *Serine/threonine-protein kinase DCLK1* |
| *D3ZQI4* | *Protein Zfand1* |
| *G3V887* | *V-type proton ATPase subunit a, Tcirg1* |
| *B1WBV0* | *Cytoplasmic tRNA 2-thiolation protein 1, Ctu1* |
| F1M6Y6 | Protein LOC298111 |
| *B5DF86* | *Component of oligomeric golgi complex 8, Cog8* |
| *Q00238* | *Intercellular adhesion molecule 1, Icam1* |
| *B2RZ44* | *N-acetyltransferase 5 (ARD1 homolog, S. cerevisiae), Naa20* |
| *G3V6C1* | *RCG24055, isoform CRA_b, Serpinb5* |
| *F1MAH6* | *Protein Cdh11* |
| *D4A208* | *SLIT-ROBO Rho GTPase-activating protein 2, Srgap2* |
| *D3ZKH3* | *Olfactory receptor, Olr402* |
| *M0R6J5* | *Protein Nom1* |
| *B1WC84* | *Canopy 4 homolog (Zebrafish), Cnpy4* |
| *Q4KLH7* | *Protein Rad21* |
| *D4A9R2* | *Protein Pkhd1l1* |
| *D3ZX21* | *Protein Ap4e1* |
| *A0A0G2K7Y2* | *Oxidation resistance protein 1, Oxr1* |
| *A0A0G2JXG3* | *Cell cycle control protein 50A, Tmem30a* |
| *D3ZSV7* | *Protein Thumpd3,* |
| *D4AD75* | *Protein Dpy19l1* |
| *Q6P742* | *Proteolipid protein 2, Plp2* |
| F7FL53 | Protein Slc43a1 |
| *D3ZWX4* | *Protein Nckipsd* |
| *D3ZJB1* | *Microfibrillar associated protein 5 (Predicted), isoform CRA_a, Mfap5* |
| *P08494* | *Matrix Gla protein, Mgp* |
| *D3ZKC9* | *Protein RGD1559904* |
| D4A533 | Protein Tapt1 |
| *B5DF62* | *Protein Pak4* |
| *B5DER2* | *Churchill domain containing 1, Churc1* |
| *D3ZDJ9* | *Bicaudal C homolog 1 (Drosophila) (Predicted), isoform CRA_b, Bicc1* |
| *B5DEG1* | *Protein Itga8* |
| *D3ZPJ1* | *Kelch-like 11 (Drosophila) (Predicted), Klhl11* |
| *Q641Z4* | *Cyclin-dependent kinase 9, Cdk9* |
| *A0A0G2K7W2* | *Aldehyde dehydrogenase, Aldh3b1* |
| *Q6TUD4* | *Protein YIPF3* |
| *A0A0G2KB15* | *Kelch domain-containing protein 10, Klhdc10* |
| *O08623* | *Sequestosome-1, Sqstm1* |
| *A0A0G2K8E5* | *Protein Ints10* |
| *M0RCH8* | *Uncharacterized protein, Rsl1d1l1* |
| *Q63187* | *Transcription elongation factor B polypeptide 3, Tceb3* |
| *Q5XIS1* | *Protein phosphatase Slingshot homolog 3, Ssh3* |
| *Q68FR2* | *Bridging integrator 2, Bin2* |
| *D3Z837* | *CDC42 binding protein kinase gamma (DMPK-like) (Predicted), Cdc42bpg* |
| *D3ZPQ3* | *Protein XAF1,* |
| *F1LN63* | *Protein Tlr3* |
| *A0A0G2KAD4* | *Protein LBH* |
| *F1M391* | *Stimulator of interferon genes protein, Tmem173* |
| *D3ZKE0* | *Protein Gnptab, Gnptab* |
| *O88801* | *Homer protein homolog 2, Homer2* |
| *F1LXS3* | *Protein Matn2* |
| *A0A0G2JTX9* | *Derlin, Derl2* |
| *A0A0G2K679* | *Protein Gtf3c2* |
