# Supplementary material for: Human extracellular microvesicles from renal tubules reverse kidney ischemia-reperfusion injury in rats
Source: PLoS One. 2018 Aug 27;13(8):e0202550. doi: 10.1371/journal.pone.0202550 (PMC6110463; doi:10.1371/journal.pone.0202550)
Supplement: S2 Table — (DOCX) [file pone.0202550.s002.docx]

| Table 2S | |
| --- | --- |
| PROTEINS PROTECTED BY CELLS FROM SUPPRESSION IN ISCHEMIA (GREY) – *OR FROM ACTIVATION IN ISCHEMIA (BLUE)* | |
| P08932 | *T-kininogen 2* |
| G3V786 | *Protein Akr1b10* |
| P02454 | *Collagen alpha-1(I) chain, Col1a1* |
| Q09128 | *1,25-dihydroxyvitamin D(3) 24-hydroxylase, mitochondrial, Cyp24a1* |
| Q7TP58 | *Phosphoglycerate mutase, Bpgm* |
| D3ZPV8 | Protein Ggct |
| D3ZTI3 | Protein Cdh24 |
