# Supplementary material for: Human extracellular microvesicles from renal tubules reverse kidney ischemia-reperfusion injury in rats
Source: PLoS One. 2018 Aug 27;13(8):e0202550. doi: 10.1371/journal.pone.0202550 (PMC6110463; doi:10.1371/journal.pone.0202550)
Supplement: S1 Appendix — Description of experimental techniques (methods). (DOCX) [file pone.0202550.s003.docx]

**METHODS**

Ethics Statement: This study was carried out in strict accordance with the recommendations in the Guide for the Care and Use of Laboratory Animals of the National Institutes of Health. The protocols were approved by the Indiana University School of Medicine Institutional Care and Use Committee. All surgery was performed under isoflurane anesthesia and all efforts were made to minimize suffering.

Human renal tubular cells: Primary human renal tubular cells were obtained from donated normal male human kidneys declined for renal transplantation, which were anonymized by the Indiana Donor Network before release to the investigators. Our research with human kidney cells from discarded normal kidneys was reviewed and determined exempted by the Institutional Review Board of Indiana University and approved by the Indiana Donor Network. The kidney cortex was digested with type 4 collagenase (Worthington, Lakewood, NJ), 6mg/dl, at 37°C for one hour. The renal tubules were separated from damaged cells and debris by centrifugation at 46rcf for three minutes and subsequently cultured in S1 medium for three days under normoxic conditions (37°C, 37% O_2_, 5% CO_2_). Each two liters of S1 medium contains F-12 HAM (10.7gm), DMEM (8.32gm), L-glutamine (0.29gm), HEPES (4.78gm), sodium pyruvate (0.11gm), phenol red (3.2mL), 7% sodium bicarbonate solution (82.8mL), pH 7.4. S1 medium was supplemented with hepatocyte growth factor (200ng/mL), epidermal growth factor (400ng/mL) (R&D Systems, Minneapolis, MN), hydrocortisone (100ug/mL), insulin (35ug/mL), transferrin (32ug/mL), sodium selenite (42ng/mL) (Sigma, St. Louis MO), fetal bovine serum at 10% and antibiotic/antimycotic solution at 1%. Cultured renal tubular cells were then either used for experiments or frozen at -80°C in IMDM medium with 30% FBS and 10% DMSO. Prior to injection, cells were transfected with lipofectamine 2000 (Invitrogen, Waltham, MA) and human pcDNA3.1-SAA1.3 and pAcGFP1-C1 plasmids, 20 ug each, in 20 ml of Opti-MEM. The transfection was designed to track the donor cells in recipient kidneys in vivo [1, 2]. The cells were labeled with primary antibodies to Human Leukocyte antigen A1 (HLA, rabbit; # ab185706, Abcam, Cambridge MA); 1:100, Pan-cytokeratin (mouse; # MA5-12231, Life technologies, Carlsbad, CA) 1:100, and organic anion transporter 1 (OAT 1, rabbit, # OAT11-A, Alpha Diagnostic International, San Antonio, TX). The secondary antibodies (all at 1:200) were from Jackson ImmunoResearch (West Grove, PA); including Texas Red-conjugated donkey anti-rabbit (# 111-075-045) and donkey anti-rabbit (green; # 711546-152).

Cellular hypoxia precondition: Given our prior finding of improved function using rat exosomes derived from hypoxia preconditioned cells [3], cells were cultured for 3 days under normoxic conditions and then transferred to previously-equilibrated hypoxic S1 medium (4h at 37°C, 1% O_2_, 5% CO_2_) in a hypoxic chamber (SCI-tive Dual, Baker Ruskinn, Sanford, ME). The cells were then cultured in oxygenated (normoxic media), exosome-free S1 medium for another 72 hours, their exosomes collected (below) and injected. The exosome originating cells were detached with trypsin, washed in S1 medium by centrifugation twice, re-suspended in PBS and injected.

Exosome collection: Human renal tubular cells from five different kidneys were plated at 30% confluence in 225cm^2^ culture flasks with S1 medium which was previously depleted of exosomes by sequential centrifugation. Cells were cultured for three to four days at 37% O_2_, 5% CO_2_, and their exosomes were collected from the medium by sequential centrifugation: 500rcf for 5min, recovering supernatant; 10,000rcf for 25min, recovering supernatant; vacuum filter with 0.2um in order to remove larger extracellular vesicles (apoptotic bodies and microvesicles); 100,000rcf for 70min, recovering the pellet. The exosome pellet was suspended in PBS. Presence of exosomes was validated by electron microscopy, nanotracker analysis, and by quantifying CD63 on immunoblots [1, 3].

Electron Microscopy and nanotrack analysis of exosomes: Exosomes released by human kidney tubular cells were identified by electron microscopy. The exosomes were fixed in 2% paraformaldehyde, 2% glutaraldehyde, 0.1M phosphate and adsorbed to a 200-400 mesh carbon/formvar-coated grid and the negative stain (Nanovan, Nanoprobes, Yaphank, NY) added. The exosomes were visualized by electron microscopy (Tecnai G2 12 Bio Twin Microscope, FEI, Hillsboro, OR) equipped with an AMT CCD camera (Advanced Microscopy Techniques, Danvers, MA). Size and number of harvested exosomes were also measured with a nanoparticle analyzer, ZetaView, from Particle Metrix in Mebane, NC. The exosome measurements were obtained by the Islet and Physiology Core of the Center for Diabetes and Metabolic Diseases at Indiana University.

Exo-Glow labeling: Human kidney tubular cells and their exosomes were labeled with Exo-Green Exo-Glow reagent (EXOG200A-1 (System Biosciences, Palo Alto CA) to track down their fusion with target cells in vivo. Briefly, cells were plated in S1 medium (detailed above) and allowed to grow to confluence for at least 24h. Cells and exosomes were collected and immediately labeled with Exo-Green, suspended in PBS, and injected in the rat tail veins.

Animal Protocols: The studies were performed on 8-week old male Nude rats (NIH-Foxn1^rnu^ or RNU rats), from Charles River Laboratories. The rats were anesthetized with inhaled isofluorane (0.5-1%) and placed on a homeothermic table to maintain a body core temperature at ~37^o^C. In three groups of rats (below) both renal pedicles were occluded for 50 minutes with microaneurysm clamps as described [1, 2].  In a fourth group, sham rats underwent an identical surgical procedure, except that the renal pedicles were not clamped.  The rats received either normal human primary renal tubular cells, 3 X 10**^6^** cells, or their exosomes (exosomes secreted over 72 hours by 3 X 10**^6^** cells: 100 ug exosomal protein, or 13.6E10 exosomes) dissolved in 0.5ml of sterile physiological saline. Cells or exosomes were injected 24 and 48 hours after ischemia via the tail vein, and two doses of each were given to increase therapeutic effectiveness. In addition, four other rats were subjected to similar renal ischemia and then injected with GFP positive human kidney cells, or with their exosomes labeled with Exo-Green Exo-Glow dye at 24 hours of reperfusion. The rats were closely monitored after injections for any evidence of pain and discomfort; and always recovered rapidly without signs of pain or distress. These rats were terminated 24 hours after injections, and injected cells or exosomes localized by fluorescence microscopy. Blood samples were obtained daily from the saphenous or tail veins and analyzed for creatinine (Pointe Analyzer, Canton MI). Rats were sacrificed at 6 days of reperfusion: following adequate anesthesia with isofluorane, organs were removed, frozen or fixed in 3.8% paraformaldehyde.

Histology and immunohistochemistry: Kidneys were fixed in 3.8% paraformaldehyde, paraffin embedded, and 5 μM sections obtained for Masson’s trichrome to stain connective tissue, and periodic acid Schiff (PAS) to image cellular morphology. The percentage of tubules in the outer medulla that showed loss of cells, luminal debris and/or tubular dilation was estimated on coded sections without the knowledge of the experimental group to which the animals belonged. Renal neutrophils were visualized with primary anti-rat neutrophil rabbit antibody (# ABIN2586050, antibodies-online.com) and secondary antibody with HRP tag (# SK-4805, Vector, Burlingame, CA). The neutrophils were counted in blinded renal sections. The areas of glomerular and peritubular fibrosis were quantified using Metamorph imaging processing software (Sunnyvale, CA) and expressed as fractional areas per 200 X microscope field, covering all available surfaces in all coded kidneys. Atrophic tubules were counted in blinded PAS stained sections. Renal sections were immuno-stained for 4-hydroxynonenal-adducts (HNE) to estimate tissue peroxidation. The primary HNE rabbit antibody was applied at 1:300 dilution (antibodies-online.com #ABIN873270). Horse anti-rabbit antibody was used to develop: ImmPRESS™ HRP Anti-Rabbit IgG (Peroxidase) Polymer Detection Kit (MP-7401, Vector). Other de-paraffinized kidney sections were incubated overnight at 4^o^C with primary goat anti-complement component 3 antibody (# c312-A, Alpha Diagnostics, San Antonio, TX). The sections were then washed 3 times in phosphate buffered saline (PBS) for 5 min, and then incubated with Texas Red-conjugated donkey anti-rabbit (# 111-075-045, Jackson ImmunoResearch) for 1 hour, followed by triple washing with PBS. Renal apoptosis was evaluated with a TUNEL assay, and apoptosis detected by antibody-linked immunohistochemistry, as recommended by the manufacturer (APO-BrdU-IHC, Bio Rad, Hercules, CA).

Microvasculature visualization by immunofluorescence: kidneys were immersion-fixed in 3.8% paraformaldehyde, followed by cryo-protection in 20% sucrose/PBS and mounted in optimal cutting temperature compound. Serial cross sections (10μm thick) were cut on a cryostat, mounted, and fixed to slides. Sections were incubated with Lycopersicon Esculentum (Tomato) Lectin, conjugated with Alexa-Fluor 594 (DL-1177, Vector) at 1:300, overnight at 4°C. DAPI (Molecular Probes, Eugene, OR) was applied at 300 nM for 5 min at room temperature. Images were taken through an inverted microscope (Zeiss Axio Imager.D2, Oberkochen, Germany). Positive fluorescence was quantified from >10 sections per animal by Aperio’s Positive Pixel Count algorithm (Aperio Technologies, Inc. Vista, CA). Negative controls for all targets were analyzed to ensure that the algorithm detected minimal false-positives and autofluorescence. Microvascular density was expressed as fractional positivity (positive pixels/total pixels on each of 400X fields).

Human Leukocyte Antigen-A1 (HLA) detection in rat kidney sections: Frozen sections were also used to visualize HLA in kidneys of rats injected with human kidney cells and their exosomes. The fixed sections were washed (above) and incubated with primary anti-HLA antibody (# ab185706, Abcam) prior to secondary antibody, as well as rhodamine (red) tagged phalloidin (# R415, Life Technologies), and Hoechst 33342 nuclear dye (1:5000), ThermoFisher, Waltham, MA.

Immuno(Western)blotting**:** Kidney cortices were homogenized in 25 mM Tris, pH 7.6, 150 mM NaCl, 1% deoxycholate, 1% P-40, 0.1% SDS, and 2X Halt Protease Inhibitor Cocktail (Thermo Scientific, Rockford, IL) and adjusted to a protein concentration of 2 mg/ml. The homogenates (20 µg) were fractionated by electrophoresis through 16.5% polyacrylamide Tris-Tricine gels. After electrophoresis, proteins were transferred to a nitrocellulose membrane. Blocking was carried out in 1% casein, 1X PBS for 1 hour. Incubation with primary antibody diluted in 1X PBS was for 1 hour. The following rabbit primary antibodies were from Abcam: anti-superoxide dismutase (1:1,000, ab52950); anti-catalase (1:2,000, ab16731); anti-erythropoietin (1:500, ab135390); anti-GLUT1 (1:250, ab652); antiHSP27 (1:1,000, ab5579); anti-actin (1:1,000, ab8227), mouse anti-actin (1:50,000, Ab6276). Anti-VEGF (1:500, sc507, and anti-CD63 (1:250 sc15363) were from Santa Cruz Biotechnology, Santa Cruz, CA. Mouse anti-PCNA (1:3,000, p8825) was from Sigma, St Louis, MO. Secondary antibodies were from Li-Cor Biosciences, Lincoln, Nebraska: IRDYE800CW Donkey anti Rabbit (green, 1:15,000, 926-32213), IRDYE 680RD Donkey anti Rabbit (red, 1:20,000, 926-68073), IRDYE800CW Donkey anti Mouse (green, 1:15,000, 926-32212), IRDYE 680RD Donkey anti Mouse (Red, 1:20,000, 926-68072), IRDYE 680RD Donkey anti Goat (red, 1:20,000, 926-68074). The membrane was then washed in 1X PBS and incubated with secondary antibodies diluted in 1X PBS for 1 hour. Secondary antibodies were IRDye 680 goat anti-rabbit IgG (1:15,000) (Li-Cor Biosciences, Lincoln, NB) and IRDye 800 CW goat anti-mouse IgG (1:20,000) (Li-Cor Biosciences). After washing in 1X PBS, the filter was scanned using an Odyssey Infrared Imaging System (Li-Cor Biosciences) for visualization of immune-reactive proteins. All steps were carried out at room temperature.

SAA1 and HLA-A1 mRNAs in kidneys: RT-PCR was used to amplify SAA1 mRNA in recipient kidneys and donor human exosomes. RNA extracted from homogenized renal cortices in lysis buffer was isolated with a purification kit as recommended by vendor (# 12183-555, ThermoFisher), and cleaned with an RNeasy Mini kit as recommended by vendor (# 74104, Qiagen, Valencia CA). For RT-PCR, 2 µg of total RNA were used to synthesize cDNA with an AffinityScript QPCR cDNA Synthesis Kit as recommended by vendor (# 600559, Aligent Technologies, Santa Clara CA). Human SAA1 mRNA was amplified as previously described (8) using the following primers selected from exon 3 of the human SAA1.3 gene [4]:

F1: 5’-TC GGC TCA GAC AAA TAC TTC C-3’ (exon 3)

R1: 5’-CTG GAT ATT CTC TCT GGC ATC G-3’ (exon 4)

The 110 bp PCR products were separated in a 2.5 % agarose gel.

Human HLA-1A was amplified using the following primers:

F1: 5'-CAT CAT TGC TGG CCT GGT TC -3'

R1: 5'-AGA AAT CTC AGA CCC CAC CCT-3'

The 119 bp PCR products were separated in a 2.5 % agarose gel.

RT-PCR was also performed on hypoxic preconditioned human renal tubular cells and their exosomes. For these experiments, we used primers specific to the following human transcripts: catalase (Cat, NM_012520 or NM_001752), hypoxia inducible factor-1a (HIF-1a, NM_024359 or NM_001530), superoxide dismutase-1 (SOD-1, NM_017050 or NM_000454), and human beta actin: (Cat, Hs.PT.39a22214847 and Rn.PT.39a22214838.g).

Renal gene expression: quantitative real-time polymerase chain reaction (qRT-PCR) was used to quantify mRNA from rat kidneys. RNA was extracted from kidneys with the RNeasy Plus mini Kit (Qiagen, Germantown MD). AffinityScript cDNA Synthesis Kit (Agilent Technologies, Santa Clara CA) was used to make cDNA from 1 ug mRNA. RNA was subsequently amplified and quantified via Mx3005P QPCR System (Agilent Technologies) along with PrimeTime Gene Expression Master Mix and PrimeTime qPCR Primer Assays (Integrated DNA Technologies, Coralville, Iowa) specific to the following rat genes: Collagen 1a (COL1A, Rn.PT.58.7562513), Transforming Growth Factor beta (TGFB, Rn.PT.58.6690138), plus complement components C1q, Rn.PT.58.36116635; C2, Rn.PT.58.13665634; and C3, Rn.PT.58.38105143.

Proteomic analysis post-ischemia and after exosome therapy: Rat kidneys were analyzed by the IUSM Proteomics Core Facility. Kidney samples were ground frozen in liquid N**_2_** and resuspended in 8 M urea/100mM Tris pH 8.5. Each sample was reduced and alkylated with tris(2-carboxyethyl)phosphine (TCEP) and chloroacetamide (CAM), digested with Trypsin Gold (Promega, Madison WI), and cleaned up with the Sep-Pak C18 purification system (Waters, Milford, MA). Samples were labeled with 6-plex or 10plex TMT Mass Tagging Reagents (Proteome Sciences, London, UK). Following TMT labeling, peptide concentration was measured using Pierce Quantitative Colorimetric Peptide Assay Kit (Pierce, Rockford, IL), and 100 ug of each sample was fractionated with a Pierce High pH Reversed-Phase Peptide Fractionation Kit. The eluted fractions were dried using a SpeedVac and re-suspended in 0.1% formic acid before LC-MS/MS analysis. Eight samples per group were analyzed on a 22cm 100μm inner diameter reverse-phase column containing 3μM ReproSil-Pur C18-Aqua (Thermo Scientific) over a 3-hour 5-35% acetonitrile gradient. The column chromatography was performed in-line with a Fusion Lumos Orbitrap mass spectrometer using a Nanoflex Easy nanospray ion source (Thermo Scientific). Data was acquired in the top speed mode with the full MS acquisition performed at a resolution of 120,000 and MS/MS analysis performed at a resolution of 50,000 to facilitate reporter ion quantitation. MS/MS fragmentation was performed by higher-energy collision dissociation (HCD) with a stepped collision energy of 30 ± 6.

MS/MS database search was performed with the relevant Uniprot FASTA database (below) using SEQUEST HT within Proteome Discoverer 2.2 (PD 2.2, Thermo Scientific). Specifically, renal data were compared to a rat database with lab-defined common contaminants including proteolytic enzymes, immunoglobulins, and bovine serum albumin. The reporter ion quantitation node within PD 2.2 was used for quantitation following sample normalization based on the total amount of protein detected for each reporter group. Data were filtered to give a false discovery rate (FDR) of ≤1% with a precursor mass tolerance of 10 parts per million (ppm) and a fragment mass tolerance of 0.6 dalton. For significance analysis, p-values were calculated using an ANOVA and a Tukey HSD post-hoc test. % with a precursor mass tolerance of 10 parts per million (ppm) and a fragment mass tolerance of 0.6 dalton. For significance analysis, p-values were calculated using an ANOVA and a Tukey HSD post-hoc test. Pathway analysis and cell compartment categorization were performed using the database for annotation, visualization and integrated discovery (DAVID) bioinformatics resources of the National Institutes of Health [5, 6] and WEB-based GEne SeT AnaLysis (WebGestault) [7, 8].

Statistics: data are expressed as mean ± SE where appropriate. Analysis of variance (post hoc via Tukey’s procedure) was used to determine if differences among mean values reached statistical significance. Student's t-test (1-tailed, 2 sample, unequal variance) was used for comparisons where only two groups were studied (GraphPad Prism, La Jolla, CA). Tukey’s procedure was used to correct for multiple comparisons.

Rat renal tubular cell hypoxia and exosome treatment: Six week-old Sprague Dawley rats (Charles River), were sacrificed by removing both kidneys under general anesthesia, and renal tubular cells were harvested from the kidneys. Kidney cortices were sliced and minced in S1 medium (below), and digested with type 4 collagenase (Worthington, Lakewood, NJ), 6 mg/dl, at 37oC in 38 % O2 and 5 % CO2 for 50 minutes. The renal tubules were then separated by percoll gradient [3], and initially cultured for 6 days in 12-well culture plates (n=5) in S1 medium: Each 2 liter contains F-12 HAM, 10.7 gm; DMEM, 8.32 gm; L-glutamine, 0.29 gm; HEPES, 4.78 gm; sodium selenite, 1.7 mg; sodium pyruvate, 0.110 gm; 3.2 ml phenol red, and pH was adjusted to 7.4 with sodium bicarbonate (Sigma). S1 medium was supplemented with hepatocyte growth factor, 200 ng/ml, and epidermal growth factor, 400 ng/ml (R&D Systems, Minneapolis, MN). The medium also contained hydrocortisone 100 ug/ml, insulin, 35 ug/ml, transferrin, 32 ug/ml, sodium selenite 42 ng/ml (Sigma, St. Louis MO), with 10 % FCS. The following day, renal tubular cells were divided into three groups: two groups were placed in a hypoxic chamber (Sci-tive Dual, Baker Ruskinn, Sanford MA) with hypoxic S1 medium (1% O**_2_**/5% CO**_2_**) for 12 hours, while the oxygenated group remained in 38% O**_2_**/5 % CO**_2_**. Subsequently, all cells was were changed to freshly oxygenated S1 culture medium (38 % O2 and 5 % CO2) and kept an additional 24 hours. The previously hypoxic groups were re-oxygenated with or without added human renal cell exosomes (15 ug exosomal protein/well). The oxygenated S1 medium was previously depleted of extracellular vesicles by 100,000 X g centrifugation twice for 90 minutes for 24 more hours. At the end of the 24 hour re-oxygenation period, all cells were incubated with 150 uM propidium iodide (red) for 5min, washed with PBS five times, and fixed with 2 % paraformaldehyde.

REFERENCES FOR METHODS SUPPLEMENT:

1. Kelly KJ, Zhang J, Han L, Kamocka M, Miller C, Gattone VH, 2nd, et al. Improved Structure and Function in Autosomal Recessive Polycystic Rat Kidneys with Renal Tubular Cell Therapy. PLoS One. 2015;10(7):e0131677. doi: 10.1371/journal.pone.0131677. PubMed PMID: 26136112; PubMed Central PMCID: PMCPMC4489886.

2. Kelly KJ, Zhang J, Han L, Wang M, Zhang S, Dominguez JH. Intravenous Renal Cell Transplantation (IRCT) with SAA1 positive cells prevents progression of chronic renal failure in rats with ischemic-diabetic nephropathy. Am J Physiol Renal Physiol. 2013;305(12):F1804-12. doi: 10.1152/ajprenal.00097.2013. PubMed PMID: 24133118.

3. Dominguez JH, Liu Y, Gao H, Dominguez JM, 2nd, Xie D, Kelly KJ. Renal Tubular Cell-Derived Extracellular Vesicles Accelerate the Recovery of Established Renal Ischemia Reperfusion Injury. J Am Soc Nephrol. 2017:3533-44. doi: 10.1681/ASN.2016121278. PubMed PMID: 28747315.

4. Yamada T, Okuda Y, Takasugi K, Wang L, Marks D, Benson MD, et al. An allele of serum amyloid A1 associated with amyloidosis in both Japanese and Caucasians. Amyloid. 2003;10(1):7-11. PubMed PMID: 12762135.

5. Huang da W, Sherman BT, Lempicki RA. Systematic and integrative analysis of large gene lists using DAVID bioinformatics resources. Nat Protoc. 2009;4(1):44-57. doi: 10.1038/nprot.2008.211. PubMed PMID: 19131956.

6. Huang da W, Sherman BT, Lempicki RA. Bioinformatics enrichment tools: paths toward the comprehensive functional analysis of large gene lists. Nucleic Acids Res. 2009;37(1):1-13. doi: 10.1093/nar/gkn923. PubMed PMID: 19033363; PubMed Central PMCID: PMCPMC2615629.

7. Wang J, Duncan D, Shi Z, Zhang B. WEB-based GEne SeT AnaLysis Toolkit (WebGestalt): update 2013. Nucleic Acids Res. 2013;41(Web Server issue):W77-83. doi: 10.1093/nar/gkt439. PubMed PMID: 23703215; PubMed Central PMCID: PMCPMC3692109.

8. Wang J, Vasaikar S, Shi Z, Greer M, Zhang B. WebGestalt 2017: a more comprehensive, powerful, flexible and interactive gene set enrichment analysis toolkit. Nucleic Acids Res. 2017;45(W1):W130-W7. doi: 10.1093/nar/gkx356. PubMed PMID: 28472511; PubMed Central PMCID: PMCPMC5570149.
